# Supplementary material for: Rescue of deficits by Brwd1 copy number restoration in the Ts65Dn mouse model of Down syndrome
Source: Nat Commun. 2022 Oct 26;13:6384. doi: 10.1038/s41467-022-34200-0 (PMC9606253; doi:10.1038/s41467-022-34200-0)
Supplement: Supplementary file 1 — Supplementary Information [file 41467_2022_34200_MOESM1_ESM.pdf]

Supplementary Information for

Rescue of deficits by *Brwd1* copy number restoration in the Ts65Dn mouse model of Down syndrome

Sasha L. Fulton#, Wendy Wenderski#, Ashley E. Lepack#, Andrew L. Eagle, Tomas Fanutza, Ryan M. Bastle, Aarthi Ramakrishnan, Emma C. Hays, Arianna Neal, Jaroslav Bendl, Lorna A. Farrelly, Amni Al-Kachak, Yang Lyu, Bulent Cetin, Jennifer C. Chan, Tina N. Tran, Rachael L. Neve, Randall J. Roper, Kristen J. Brennand, Panos Roussos, John C. Schimenti, Allyson K. Friedman, Li Shen, Robert D. Blitzer, Alfred J. Robison, Gerald R. Crabtree, Ian Maze*

Corresponding author. Email: ian.maze@mssm.edu
#These authors contributed equally to this work

This file includes:

Extended Captions for Figures 1 to 4
Supplementary Figures 1 to 14
Supplemental Figure 15 (uncropped blots)

Other Supplementary Material for this manuscript includes the following:

Description of Additional Supplementary Files
Supplementary Data 1 to 10

Extended captions for Figures 1 to 4

Figure 1. ***Brwd1*** **copy number restoration rescues synaptic and cognitive deficits in male trisomic animals.**

(**a**) qPCR expression data for *Brwd1* in embryonic day (E) 17.5 forebrain (FB; *n* = 11 euploid *vs.* 7 Ts65Dn) and adult (6-week) PFC (*n* = 9 euploid *vs.* 5 Ts65Dn), hippocampus (HIPP; *n* = 9 euploid *vs.* 5 Ts65Dn) and cerebellum (CER; *n* = 9 euploid *vs.* 5 Ts65Dn) from euploid *vs.* Ts65Dn male mice. Student's two-tailed t-test [E17.5 FB: $t_{16} = 2.476$, *p = 0.0249; PFC: $t_{12} = 5.912$, ****p<0.0001; HIPP: $t_{12} = 2.307$, *p = 0.0397; CER: $t_{12} = 2.872$, *p = 0.0140]. (**b**) Schematic depicting the generation of mouse genotypes to be investigated: euploid *vs.* *Brwd1*$^{+/-}$ *vs.* Ts65Dn *vs.* Ts65Dn*;Brwd1*$^{+/-}$. (**c**) qPCR expression data for *Brwd1* in euploid (*n* = 12) *vs.* *Brwd1*$^{+/-}$ (*n* = 12) *vs.* Ts65Dn (*n* = 11) *vs.* Ts65Dn*;Brwd1*$^{+/-}$ (*n* = 11) E17.5 forebrain. One-way ANOVA with Tukey's post-hoc analyses [$F_{3,42} = 30.06$, p<0.0001; Tukey's – **p<0.01, ***p<0.001, ****p<0.0001]. (**d**) Deficiency of hippocampal LTP in male Ts65Dn mice is rescued by *Brwd1* copy number restoration. Over the final 5 min of recording, LTP was reduced in slices from Ts65Dn mice (*n* = 7 slices from 3 mice) compared to euploid (*n* = 8 slices from 3 mice), *Brwd1*$^{+/-}$ (*n* = 9 slices from 3 mice) and Ts65Dn*;Brwd1*$^{+/-}$ mice (*n* = 9 slices from 3 mice), which in turn did not differ from one another. One-way ANOVA [$F_{3,29} = 8.436$, p=0.0003; Tukey's – *p<0.05, **p<0.01, ***p<0.001]. The representative traces were recorded at the end of the baseline period (dashed lines) and 60 min after induction of LTP (solid lines). Calibrations: 0.5 mV / 5 ms. (**e**) Context dependent fear conditioning – displayed as % freezing in the trained context – comparing euploid (*n* = 16) *vs.* *Brwd1*$^{+/-}$ (*n* = 13) *vs.* Ts65Dn (*n* = 12) *vs.* Ts65Dn*;Brwd1*$^{+/-}$ mice (*n* = 8). One-way ANOVA with Dunnett's post-hoc analyses vs. Ts65Dn [$F_{3,45} = 3.516$, p=0.0225; Dunnett's – *p<0.05]. Data are presented as averages ± SEM. Source data are provided as a source data file.

Figure 2. Rescue of aberrant gene expression in male trisomic brain by *Brwd1* renormalization.

(a) RNA-seq heatmaps (ordered via unsupervised clustering) of DE genes (FDR<0.1) comparing euploid (n = 8) *vs.* Ts65Dn (n = 7) *vs.* Ts65Dn*;Brwd1*$^{+/-}$ (n = 8) adult male (6-week) hippocampus. (**b**) Normalized heatmaps of RNA expression values for DE genes in adult male hippocampus that display pairwise significant regulation between Ts65Dn *vs.* euploid, and are rescued in Ts65Dn*;Brwd1*$^{+/-}$ *vs.* Ts65Dn. **(c)** Heatmap displays Jaccard index as well as adjusted p-values from odds ratio analyses of the overlap between DE genes from euploid *vs.* Ts65Dn *vs.* Ts65Dn*;Brwd1*$^{+/-}$ comparisons and previously published human DS single-nuclei RNA-seq data *vs.* age-matched controls. **(d)** Bar graph of -log10(adj. p-val) for gene ontology (GO) processes displaying enrichment for PCGs identified in (c) above (i.e., 167 rescued PCGs). Source data are provided as a source data file.

Figure 3. BRWD1 tightly associates with the BAF complex in euploid brain.

(a) Schematic of mouse brain soluble nuclear protein extract (NE) preparation, density sedimentation of nuclear proteins over a 10-30% glycerol gradient, and immunoprecipitation of BAF chromatin remodeling complexes. Blue lettering indicates neuronal-specific BAF subunits. Red lettering indicates PBAF-specific subunits. **(b)** Density sedimentation of adult *Brwd1*$^{FLAG\text{-}HA}$ brain NE over a 10-30% glycerol gradient indicates that BRWD1 predominantly associates with large protein complexes. Subunits of BAF and AP-1 complexes serve as molecular weight markers: SMARCA2/4 antibody indicates all BAF complexes including non-canonical GBAF (~1 MDa), canonical BAF (~2 MDa) and Polybromo-containing BAF (PBAF, ~3 MDa); ACTL6B and SS18L1 indicate neuronal-specific BAF complexes; c-Jun indicates AP-1 (160-440 kDa). HA signal at the expected molecular weight of BRWD1-FLAG-HA (~260 kDa) is observed in

fractions containing the BAF complex. **(c)** Endogenous BRWD1-FLAG-HA interacts with BAF complexes in embryonic brain. BAF complexes were immunoprecipitated from *Brwd1*$^{FLAG\text{-}HA}$ brain NE with antibodies against the BAF core ATPase SMARCA4, the neural progenitor subunit SS18, the neuronal subunit SS18L1 or IgG as a control. Endogenous BRWD1-FLAG-HA robustly co-immunoprecipitated with SMARCA4 and the neural progenitor subunit SS18, but less so with the neuronal subunit SS18L1 from E17.5 brain. (**d**) BAF complexes purified from adult *Brwd1*$^{FLAG\text{-}HA}$ brain NE with antibodies against SMARCA4 or the neuronal subunit SS18L1 co-immunoprecipitate BRWD1-FLAG-HA. Experiments in b-d were repeated at least 2X (technical replicates) with similar results. **(e)** The stability of the BAF:BRWD1-FLAG-HA interaction was challenged with increasing concentrations (0.25-4 M) of the denaturing agent, urea. A fraction of BRWD1 remained bound to BAF in up to 4 M urea, surpassing the stability of the dedicated BAF subunit, SMARCB1. **(f)** Quantification of urea denaturation experiments, as shown in e, with the amount of bound protein normalized to the amount of immunoprecipitated SMARCA4 (n=3 experiments). Data are presented as averages ± SEM. Source data are provided as a source data file. See **Supplemental Figure 15** for uncropped blots with MW markers.

Figure 4. *Brwd1* renormalization partially rescues genomic BAF complex mistargeting in male trisomic brain.

(**a**) Heatmaps of normalized SMARCA2/4 enrichment in euploid *vs.* Ts65Dn *vs.* Ts65Dn;*Brwd1*$^{+/-}$ adult male hippocampus (*n*=4/group) centered (–/+ 5 kb) over sites of differential SMARCA2/4 enrichment comparing Ts65Dn *vs.* euploid mice (diffReps, p<0.0001). (**b**) Volcano plot depicting regulation of SMARCA2/4 enriched PCGs (3,563) in euploid animals displaying differential enrichment in Ts65Dn mice (2,156/3,563; ~60.5%); gray circles = unregulated PCGs. Of the 2,156 PCGs regulated with respect to SMARCA2/4 enrichment, 595 are rescued (red circles; ~27.6%)

in Ts65Dn*;Brwd1*$^{+/–}$ mice, whereas the remainder of PCGs do not display such rescue (black circles). Note that many PCGs are assigned multiple Log2FCs and p-values in diffReps analyses. Therefore, for each PCG, we plotted the Log2FC (and respective p-value) for the most significant p-value/PCG. (**c**) Relative frequency (observed/expected overlap in base pairs) of each chromatin state within significant differentially enriched sites for SMARCA2/4 (Ts65Dn vs. euploid). Chromatin states were obtained from brain regions included in the Roadmap Epigenomics Project. (**d**) Bubble plots of GO processes (burgundy) and KEGG pathways (purple) displaying enrichment for rescued differentially enriched PCGs identified in (b) above (i.e., 595 rescued PCGs). **(e)** Odds ratio analysis of overlapping differentially accessible sites in Ts65Dn *vs.* euploid animals and Ts65Dn*;Brwd1*$^{+/–}$ animals, separated by direction of regulation. **(f)** Odds ratio analysis of overlapping PCGs displaying rescued differential SMARCA2/4 enrichment in Ts65Dn *vs.* euploid animals (595 from B above) *vs.* PCGs displaying differential chromatin accessibility (neuronal ATAC-seq, *n*=3/group, diffReps, p<0.0001) in Ts65Dn *vs.* euploid mice that are either rescued, or not, in their differential accessibility in Ts65Dn*;Brwd1*$^{+/–}$ animals. Insert numbers indicate respective p values for associations, followed by the number of PCGs overlapping per category. Source data are provided as a source data file.

Supplementary Figures 1 to 14

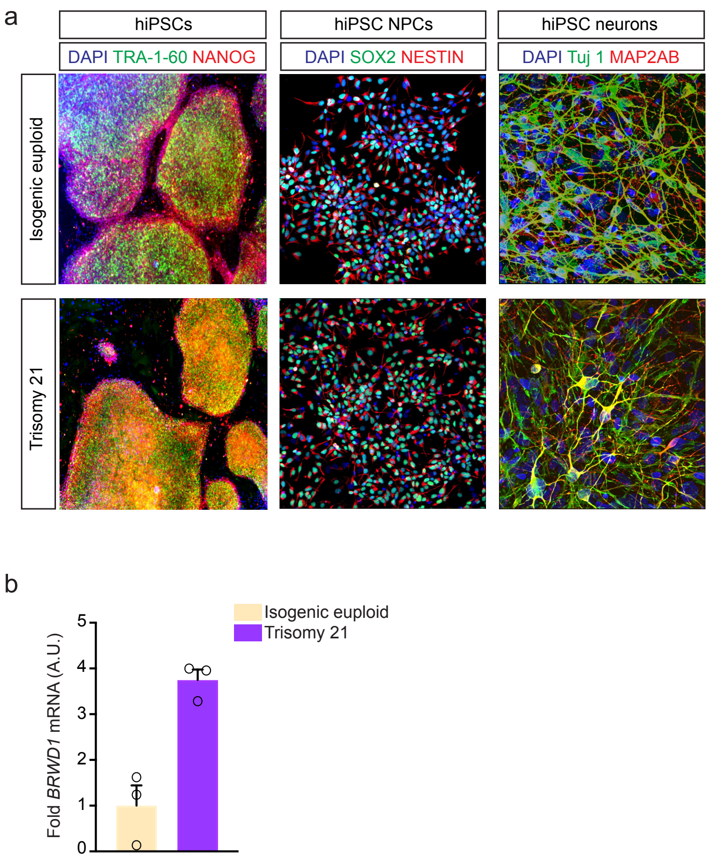

Supplementary Figure 1. *BRWD1* is overexpressed in human Trisomy 21 neurons.

(a) Trisomy 21 patient-specific hiPSCs, NPCs and neurons *vs.* those from an isogenic euploid control. Left-hiPSCs express NANOG (red) and TRA-1-60 (green). DAPI (blue). Center-hiPSC neural progenitor cells (NPCs) express NESTIN (red) and SOX2 (green). DAPI (blue). Right-hiPSC neurons express Tuj 1 (green) and the dendritic marker MAP2AB (red). DAPI (blue). Experiment repeated 3 times. **(b)** qPCR expression data for *BRWD1* in hiPSC neurons derived from a single patient with Trisomy 21 *vs.* an isogenic control. Samples were run in technical (by plate) triplicates and are thus quantified but not statistically compared. Data are presented as averages ± SEM. Source data are provided as a source data file.

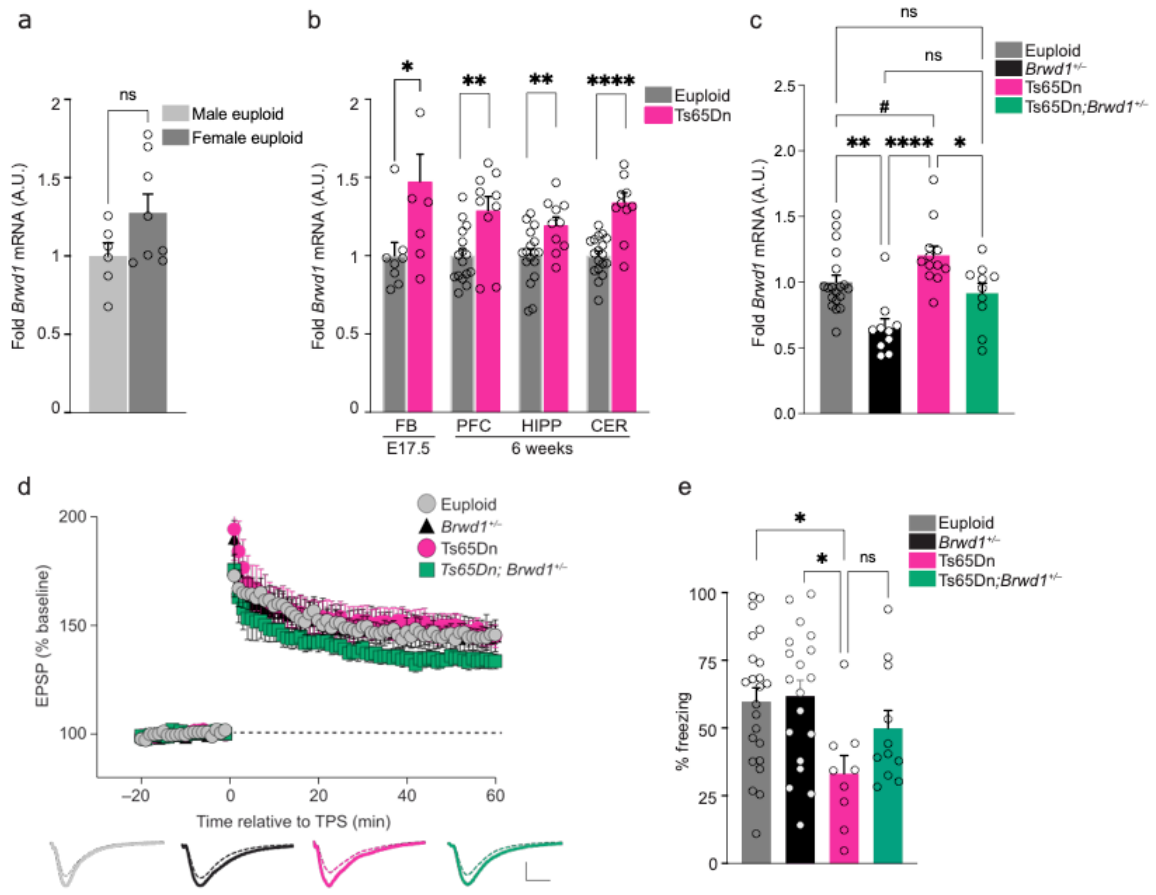

Supplementary Figure 2. Female Ts65Dn mice show distinct physiological and *Brwd1* expression phenotypes *vs.* male Ts65Dn with more modest involvement of *Brwd1*.

(**a**) qPCR expression data for *Brwd1* in male *vs.* female euploid adult hippocampus (n = 6 male *vs.* 8 female). (**b**) qPCR expression data for *Brwd1* in embryonic day (E) 17.5 forebrain (FB; n = 17 euploid *vs.* 10 Ts65Dn) and adult (6-week) PFC (n = 10 euploid *vs.* 18 Ts65Dn), hippocampus (HIPP; n = 10 euploid *vs.* 17 Ts65Dn) and cerebellum (CER; n = 18 euploid *vs.* 10 Ts65Dn) from euploid *vs.* Ts65Dn female mice. Student's t-test [E17.5 FB: t_{14} = 2.453, *p = 0.0279; PFC: t_{24}= 3.269, ** = 0.0032; HIPP: t_{25} = 2.82, ** = 0.0093; CER: t_{26}= 5.713, **** < 0.0001]. **(c)** qPCR expression data for *Brwd1* in euploid (n = 19) *vs.* *Brwd1*$^{+/-}$ (n = 10) *vs.* Ts65Dn (n = 12) *vs.* Ts65Dn*;Brwd1*$^{+/-}$ (n = 10) in adult (6wk) female hippocampus. One-way ANOVA with Tukey's post-hoc analyses [$F_{3,47}$ = 10.55, p<0.0001; Tukey's – #p = 0.0669, *p = 0.0287, **p = 0.0021,

****p<0.0001]. **(d)** In females, LTP in *Ts65Dn* mice (*n* = 8 slices from 3 mice) did not differ from that of euploid controls (*n* = 8 slices from 3 mice); p > 0.9 for each of the final 5 time points. Similarly, no significant differences were found between either of these genotypes and the *Brwd1*$^{+/-}$ (*n* = 9 slices from 3 mice) or Ts65Dn;*Brwd1*$^{+/-}$ mice (*n* = 9 slices from 3 mice); all p's > 0.14 (two-way RM ANOVA with Tukey's post-hoc analyses). Representative traces for each genotype were obtained at the end of the baseline period (dashed lines) and 60 min after induction of LTP (solid lines). Calibrations: 0.5 mV / 5 ms. **(e)** Conditioned fear responses (displayed as % freezing within the trained context) in euploid (*n* = 23) *vs.* *Brwd1*$^{+/-}$ (*n* = 19) *vs.* Ts65Dn (*n* = 9) *vs.* Ts65Dn;*Brwd1*$^{+/-}$ (*n* = 11) female mice. One-way ANOVA [$F_{3,58}$ = 3.559, p = 0.0196] with Dunnett's post-hoc analyses (*vs.* Ts65Dn); Ts65Dn *vs.* WT: *p = 0.0148, Ts65Dn *vs.* *Brwd1*$^{+/-}$: *p = 0.0105, Ts65Dn *vs.* Ts65Dn;*Brwd1*$^{+/-}$: ns = 0.2511 . Data are presented as averages ± SEM. Source data are provided as a source data file.

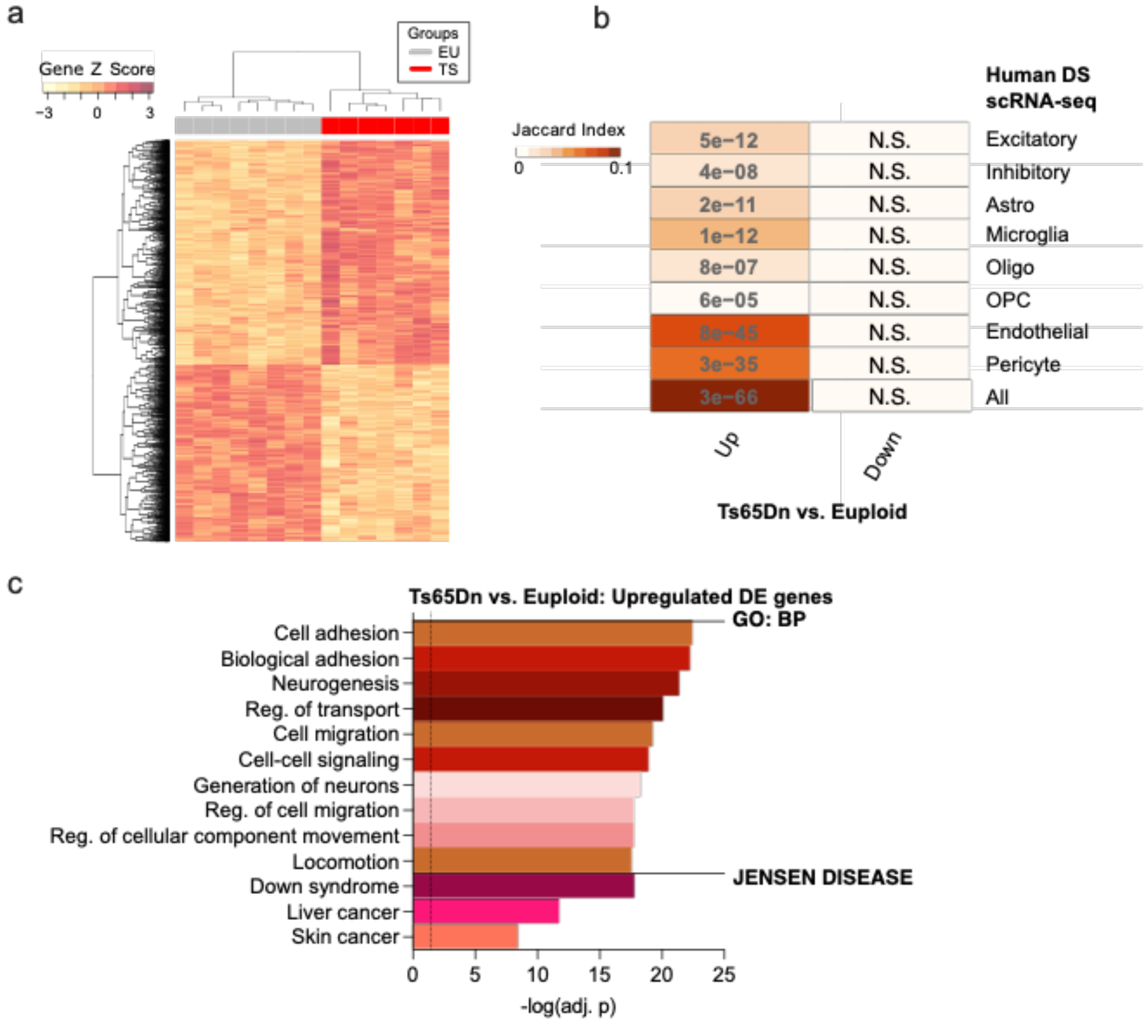

Supplementary Figure 3. Gene expression changes in embryonic E17.5 Ts65Dn forebrain significantly overlap with human DS.

(a) RNA-seq heatmaps of DE genes (FDR <.1) between e17.5 Ts65Dn (*n* = 7) *vs.* euploid (*n* = 8) forebrain tissue. Normalized RNA expression values (averaged between replicates) were used to generate z-scores for each row. **(b)** Odds ratio analyses showing significant overlap between DE genes in (a) and human DS RNA-seq profiles. **(c)** Bar graphs of -log10(adj. p-val) for gene ontology (GO) processes and Jensen disease pathways displaying enrichment for DE genes identified in (a). Source data are provided as a source data file.

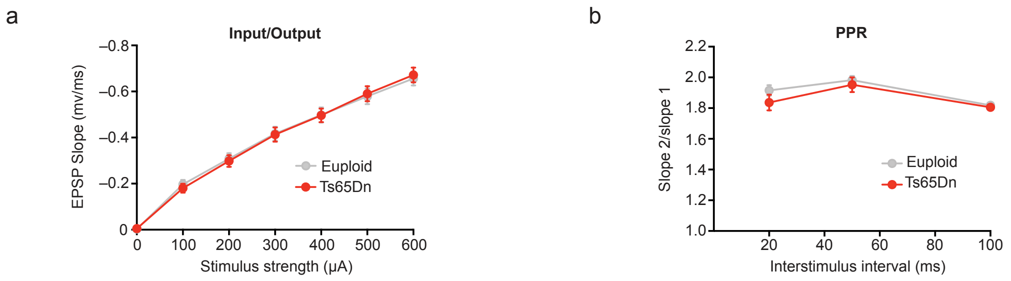

Supplementary Figure 4. Rescue of neuronal physiology deficits by *Brwd1* copy number restoration are specific to LTP.

(a) Basal synaptic efficiency at Schaffer collateral inputs to area CA1, as assessed by the input-output relationship, was not affected in Ts65Dn mice (*n* = 8 slices from 2 male mice) compared to euploid controls (*n* = 6 slices from 2 male mice); two-way RM ANOVA, effect of genotype – p = 0.98. **(b)** Paired-pulse ratio, assessed at intervals from 20 to 100 ms, did not differ between Ts65Dn mice (*n* = 8 slices from 2 male mice) and euploid controls (*n* = 6 slices from 2 male mice); two-way RM ANOVA, effect of genotype – p = 0.38. Data are presented as averages ± SEM. Source data are provided as a source data file.

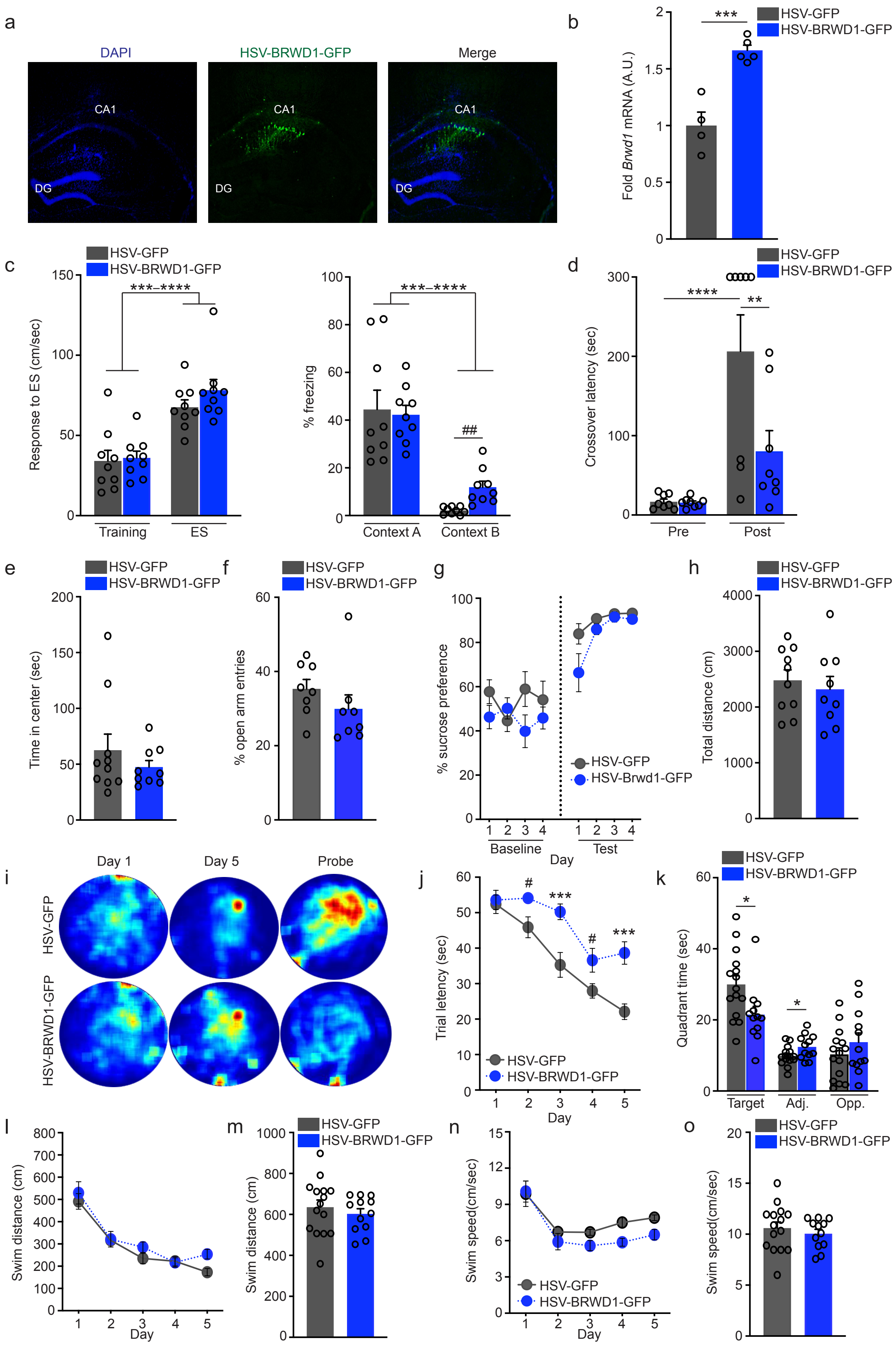

a
DAPI
HSV-BRWD1-GFP
Merge
CA1
DG
b
HSV-GFP
HSV-BRWD1-GFP
***
Fold Brwd1 mRNA (A.U.)
c
Response to ES (cm/sec)
–*
Training
ES
% freezing
##
Context A
Context B
d
Crossover latency (sec)
****
**
Pre
Post
e
Time in center (sec)
f
% open arm entries
g
% sucrose preference
HSV-Brwd1-GFP
Baseline
Day
Test
h
Total distance (cm)
i
Day 1
Day 5
Probe
j
Trial latency (sec)
#
*
k
Quadrant time (sec)
Target
Adj.
Opp.
l
Swim distance (cm)
m
n
Swim speed(cm/sec)
o

Supplementary Figure 5. *Brwd1* overexpression in adult male hippocampus is sufficient to induce cognitive deficits.

(**a**) Immunofluorescence images of mouse dorsal hippocampus (CA1) transduced with an HSV vector expressing BRWD1$^{FLAG\text{-}HA}$-GFP overlaid with a nuclear co-stain (DAPI) at 20x objective. DG = dentate gyrus. **(b)** qPCR expression data for *Brwd1* in CA1 transduced with HSV-BRWD1-GFP (*n* = 5) *vs.* HSV-GFP (*n* = 4). Student's unpaired t-test [$t_7 = 5.670$, ***p = 0.0008]. **(c)** Context dependent fear conditioning. *Left* – training *vs.* response to electric shock (ES) in context A; two-way ANOVA with Sidak's post-hoc analyses: effect of ES [$F_{1,32} = 45.60$, p<0.0001; Sidak's – GFP (***p = 0.004), BRWD1-GFP (****p<0.0001)]; effect of virus [$F_{1,32} = 1.252$, p = 0.27]. *Right* – fear conditioned responses in context A *vs.* B (displayed as % freezing); two-way ANOVA with Sidak's post-hoc analyses: effect of context [$F_{1,32} = 60.73$, p<0.0001; Sidak's – GFP (****p<0.0001), BRWD1-GFP (***p = 0.0004)]. *a posteriori* Student's t-tests comparing HSV-BRWD1-GFP *vs.* HSV-GFP in contexts A and B were performed – context B ($t_{16} = 3.861$, $^{\#\#}$p = 0.0014). No differences were observed in the conditioned context A. *n* = 9/viral group. **(d)** TDPA – two-way ANOVA with Sidak's post-hoc analyses: effect of training (pre/post) [$F_{1,28} = 23.03$, p<0.0001; Sidak's – GFP (****p<0.0001), BRWD1-GFP (p = 0.18)]; effect of virus [$F_{1,28} = 5.752$, p = 0.02; Sidak's – pre BRWD1-GFP *vs.* GFP (p = 0.9995), post BRWD1-GFP *vs.* GFP (**p = 0.0045)] *n* = 8/viral group. **(e)** Open field test (time in center) – student's unpaired t-test (p = 0.36). *n* = 10 GFP *vs.* 9 BRWD1. **(f)** EPM (% open arm entries) – student's unpaired t-test (p = 0.25). *n* = 8/viral group. **(g)** Sucrose preference (displayed as % preference over days) – analyzed as total preference baseline *vs.* test; two-way ANOVA with Sidak's post-hoc analyses [Sidak's – baseline BRWD1-GFP *vs.* GFP (p = 0.06), test BRWD1-GFP *vs.* GFP (p = 0.17)]. *n* = 8/viral group. **(h)** Locomotion (total distance) – student's unpaired t-test (p = 0.58). *n* = 10 GFP *vs.* 9 BRWD1. **(i)** MWM – representative heatmaps of movement in the maze on days 1 and 5 of

training, as well as during the probe test for both viral groups. **(j)** MWM training (trial latency to locate the platform), days 1-5. Two-way RM ANOVA with Sidak's post-hoc analyses: effects of day [$F_{4,125} = 28.21$, $p<0.0001$] and virus [$F_{4,125} = 33.49$, $p<0.0001$]; Sidak's BRWD1-GFP *vs.* GFP – day 3 (***p = 0.0008), day 5 (***p = 0.0002). *a posteriori* student's t-tests – days 2 and 4 ($^{\#}p<0.05$). *n* = 15 GFP *vs.* 12 BRWD1. **(k)** MWM probe text (quadrant time; target *vs.* adjacent/Adj. *vs.* opposite/Opp.) – student's t-tests: target ($t_{25} = 2.427$, *p = 0.02), Adj. ($t_{25} = 2.152$, *p = 0.04), Opp. ($t_{25} = 1.110$, p = 0.28). *n* = 15 GFP *vs.* 12 BRWD1. **(l-o)** Swim distance during training **(l)** and during the probe test **(m)**, as well as swim speed during training **(n)** and during the probe test **(o)** were unaffected (p>0.05 for all comparisons) by viral transduction with BRWD1-GFP; assessed via two-way RM ANOVA (Sidak's post-hoc) and student's t-tests for L/N and M/O, respectively. *n* = 15 GFP *vs.* 12 BRWD1. All viral manipulation experiments were performed in male mice. Data are presented as averages ± SEM. Source data are provided as a source data file.

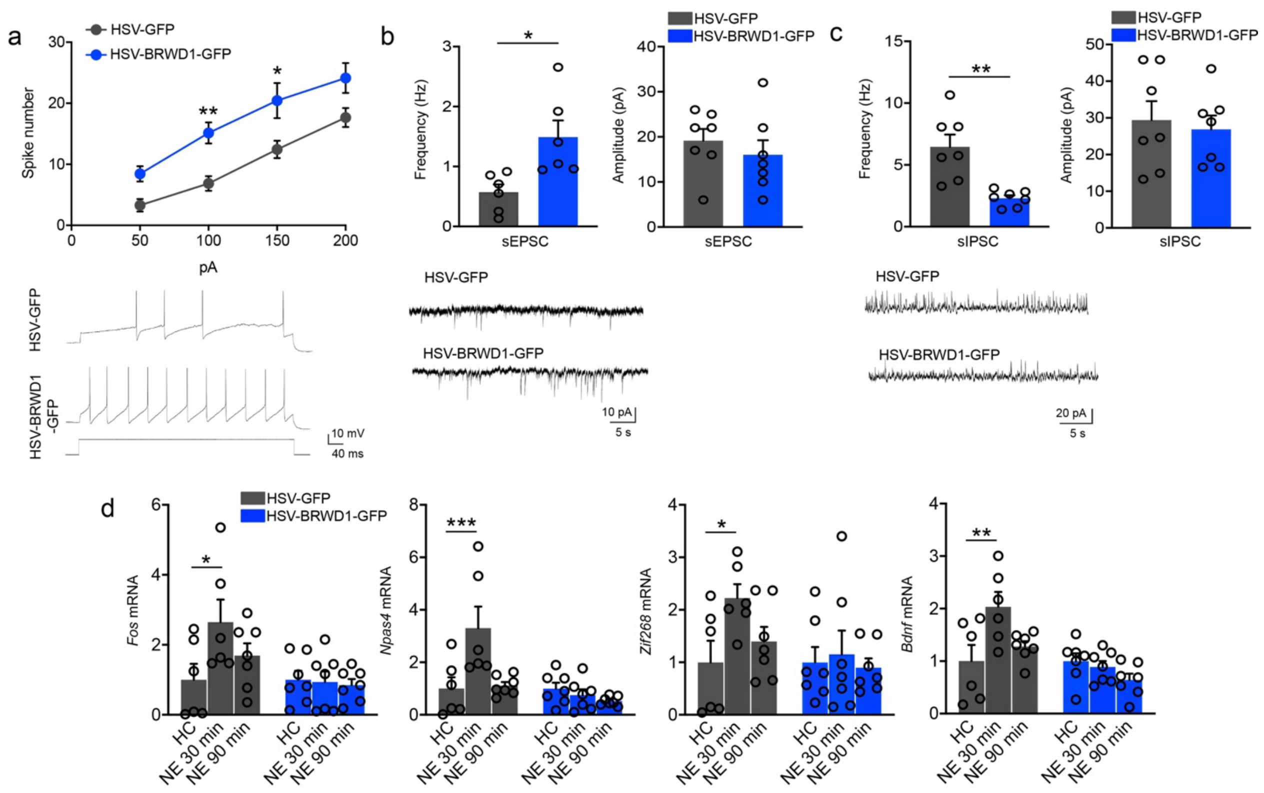

Supplementary Figure 6. *Brwd1* overexpression in adult male hippocampus inhibits transcriptional plasticity via potentiation of excitation/inhibition imbalance.

(**a**) Quantification of current-induced spike number in HSV-GFP *vs.* HSV-BRWD1-GFP expressing CA1 pyramidal neurons indicating that *Brwd1* overexpression results in increased cellular excitability (*n* =7 neurons from 3 mice/group). Student's unpaired t-test [100 pA: t_{12} = 3.940, **p = 0.0020; 150 pA: t_{12} = 2.490, *p = 0.0284]. Representative traces are provided (the bottom trace indicates the injection of 100 pA depolarizing current). (**b-c**) Representative traces and quantification of (**b**) sEPSC and (**c**) sIPSC frequencies (left) and amplitudes (right) from adult hippocampal CA1 pyramidal neurons transduced with HSV-GFP *vs.* HSV-BRWD1-GFP (*n* =7 neurons from 3 mice/group). Student's unpaired t-test [sEPSC frequency: t_{10} = 2.988, *p = 0.0136; sIPSC frequency: t_{12} = 4.058, **p = 0.0016]. (**d**) qPCR expression data for IEGs in CA1 transduced with HSV-BRWD1-GFP (*n* = 7/condition) *vs.* HSV-GFP (*n* = 6 for home cage and 30 min, *n* = 7

for 90 min); data are normalized to respective HC controls within each viral group. Two-way ANOVA with Dunnett's post-hoc analyses [*Fos:* effect of virus, $F_{1,34} = 7.705$, p=0.0089, Dunnett's – *p=0.0108; *Npas4:* effect of virus, $F_{1,34} = 11.90$, p=0.0015, effect of novel environment (NE), $F_{2,34} = 6.118$, p=0.0054, interaction, $F_{2,34} = 6.344$, p=0.0046, Dunnett's – ***p=0.0004; *Zif268:* effect of virus, $F_{1,34} = 3.901$, p=0.0564, Dunnett's – *p=0.0295; *Bdnf:* effect of virus, $F_{1,34} = 15.58$, p=0.0004, effect of NE, $F_{2,34} = 4.543$, p=0.0178, interaction, $F_{2,34} = 4.723$, p=0.0155, Dunnett's – **p=0.0012. Data are presented as averages ± SEM. Source data are provided as a source data file.

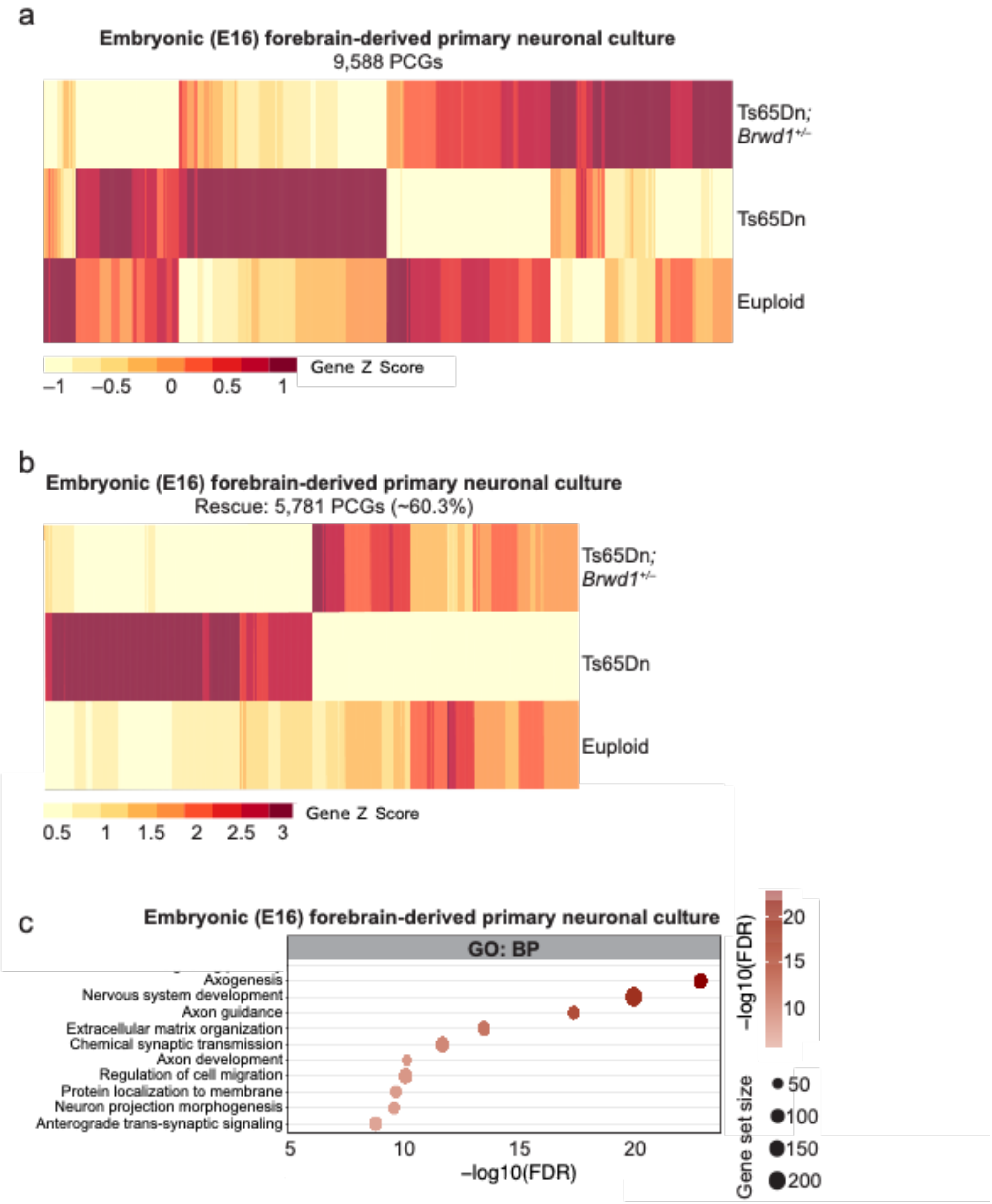

Supplementary Figure 7. Rescue of aberrant gene expression in trisomic embryonic forebrain-derived neurons by *Brwd1* renormalization.

(a) RNA-seq heatmaps of DESeq2 (likelihood-ratio-test) significant PCGs comparing euploid *vs.* Ts65Dn *vs.* Ts65Dn;*Brwd1*$^{+/–}$ E16 forebrain-derived primary neuronal cultures (mixed male and female, *n* = 3/group). **(b)** RNA-seq heatmaps of DESeq2 significant PCGs in E16 forebrain-derived primary neuronal cultures that display pairwise significant regulation between Ts65Dn *vs.*

euploid, along with pairwise significant rescue in Ts65Dn*;Brwd1*$^{+/-}$ *vs.* Ts65Dn. **(c)** Bubble plot of gene ontology (GO) processes displaying enrichment for PCGs identified in (b) above. Source data are provided as a source data file.

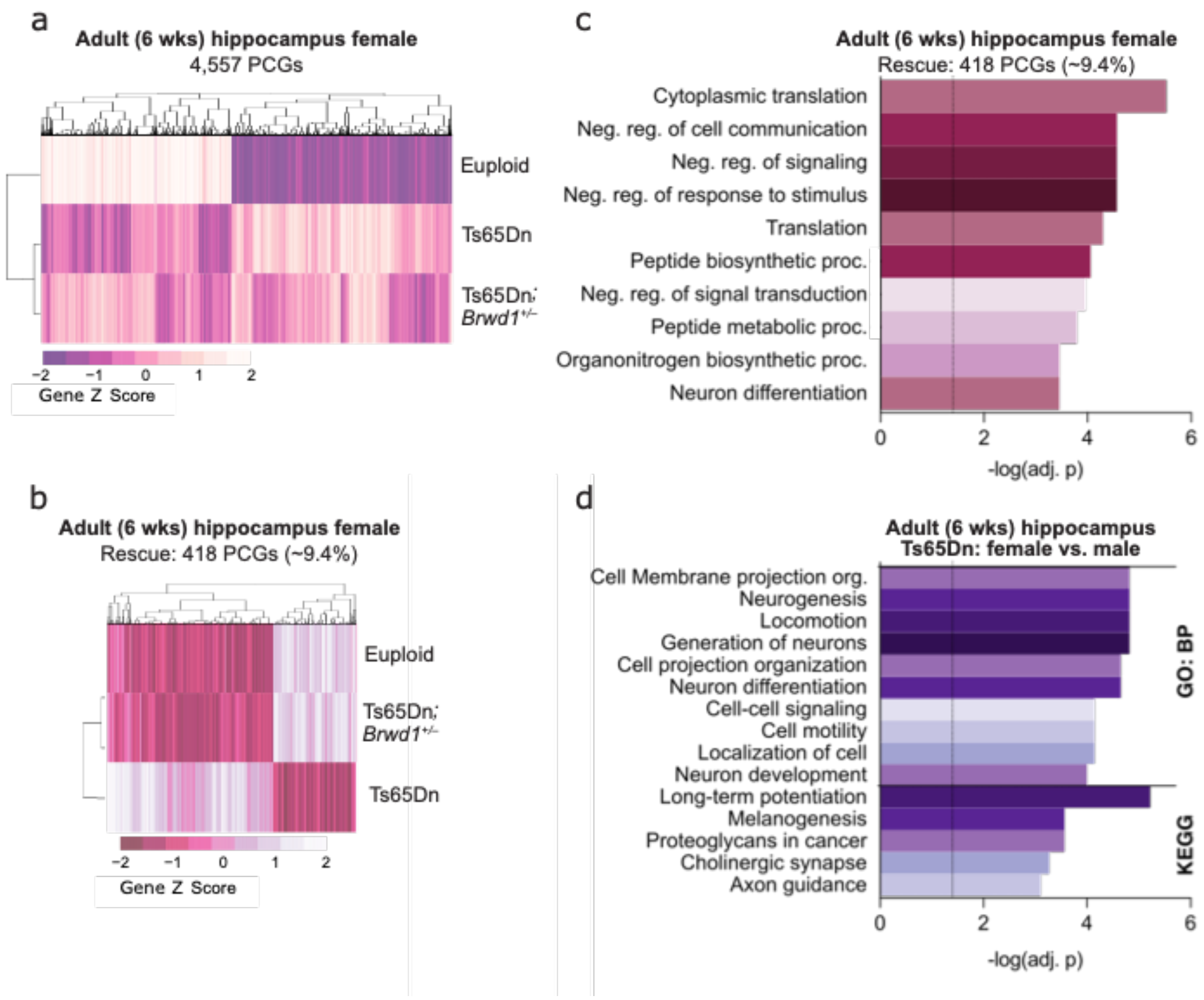

Supplementary Figure 8. RNA-seq in female Ts65Dn *vs.* euploid *vs.* Ts65Dn;*Brwd1*$^{+/-}$ mice reveals modest rescue of gene expression changes.

(a) RNA-seq heatmaps of DE genes (FDR <.1) by pairwise comparison of female euploid *vs.* Ts65Dn *vs.* Ts65Dn;*Brwd1*$^{+/-}$ adult (6-week) hippocampus (*n* = 8/group). Normalized RNA expression values (averaged between replicates) were used to generate z-scores for each row. (**b**) Normalized heatmaps of RNA expression values for DE genes in adult female hippocampus that display pairwise significant regulation between Ts65Dn *vs.* euploid, and are rescued in Ts65Dn;*Brwd1*$^{+/-}$ *vs.* Ts65Dn. **(c)** Bar graph of -log10(adj. p-val) for gene ontology (GO) processes displaying enrichment for PCGs identified in (c) above. **(d)** Bar graph of -log10(adj. p-

val) for gene ontology (GO) processes and KEGG pathways displaying enrichment for DE genes identified in between Ts65Dn female *vs.* Ts65Dn male mice. Source data are provided as a source data file.

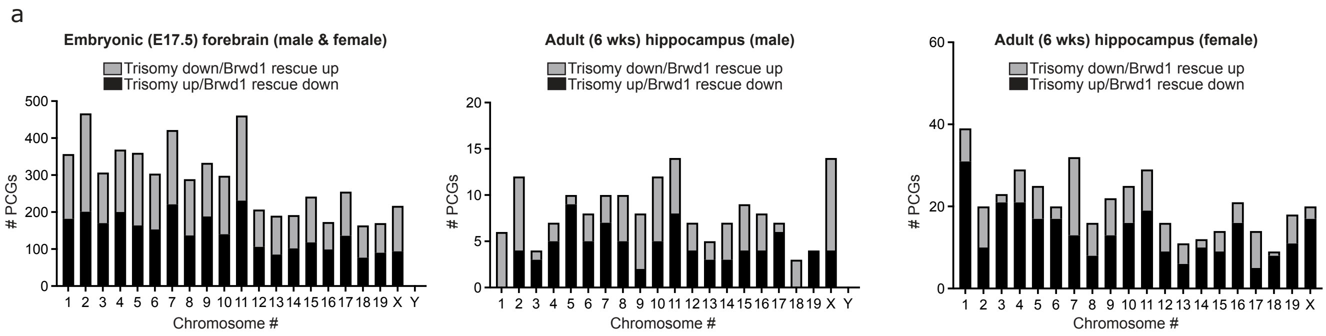

Supplementary Figure 9. Rescue of aberrant gene expression in trisomic hippocampus by *Brwd1* copy number restoration.

(a) Chromosomal enrichment of differentially expressed genes in Ts65Dn *vs.* euploid that are rescued in Ts65Dn;*Brwd1*$^{+/-}$ *vs.* Ts65Dn in (left) embryonic/E17.5 forebrain, (middle) adult male hippocampus and (right) adult female hippocampus. Source data are provided as a source data file.

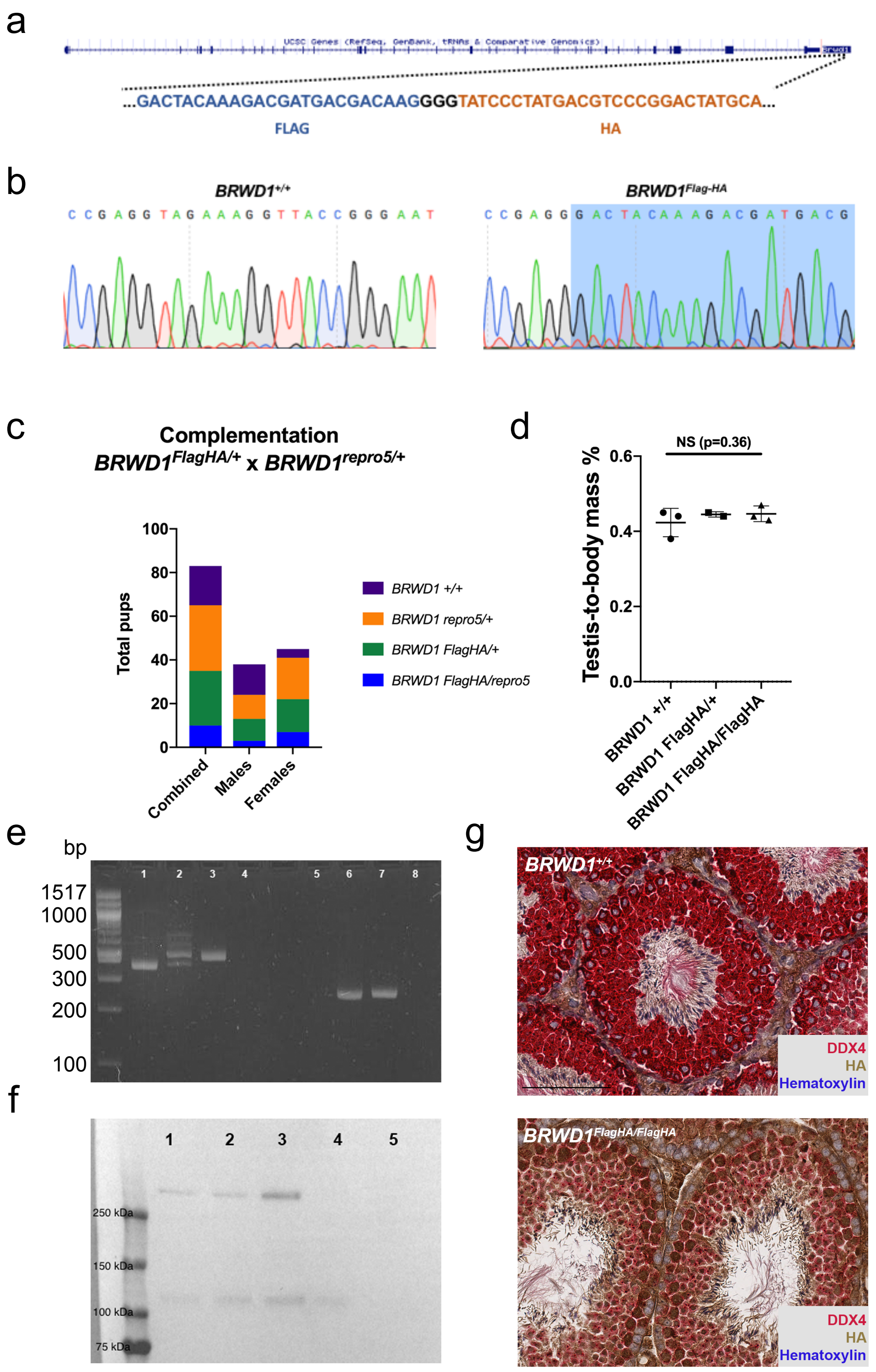

a
...GACTACAAAGACGATGACGACAAGGGGTATCCCTATGACGTCCCGGACTATGCA...
FLAG
HA
b
BRWD1+/+
CCGAGGTAGAAAGGTTACCGGGAAT
BRWD1Flag-HA
CCGAGGGACTACAAAGACGATGACG
c
Complementation
BRWD1FlagHA/+ x BRWD1repro5/+
Total pups
100
80
60
40
20
0
Combined
Males
Females
BRWD1 +/+
BRWD1 repro5/+
BRWD1 FlagHA/+
BRWD1 FlagHA/repro5
d
NS (p=0.36)
Testis-to-body mass %
0.6
0.4
0.2
0.0
BRWD1 +/+
BRWD1 FlagHA/+
BRWD1 FlagHA/FlagHA
e
bp
1517
1000
500
300
200
100
1 2 3 4 5 6 7 8
f
1 2 3 4 5
250 kDa
150 kDa
100 kDa
75 kDa
g
BRWD1+/+
DDX4
HA
Hematoxylin
BRWD1FlagHA/FlagHA
DDX4
HA
Hematoxylin

Supplementary Figure 10. Generation and validation of *Brwd1*$^{FLAG\text{-}HA}$ mice.

(a) Screenshot of the *Brwd1* sequence from the UCSC Genome Browser. The epitope sequence of 1xFLAG-GGG-1xHA was added to the C-terminus of the last exon. **(b)** Example chromatograms of wildtype *vs.* *Brwd1*$^{Flag\text{-}HA}$ mice. **(c)** Genetic complementation analyses demonstrate that *Brwd1*$^{Flag\text{-}HA}$ fully complements the null allele (*Brwd1*^{repro5}). **(d)** Relative testis:body mass of epitope-tagged animals are comparable to wildtype mice. N = 2-3/genotype. One-way ANOVA comparing the three genotypes did not reveal significant differences (p = 0.36) **(e)** *Brwd1* transcript levels in testes. Lanes 1-4 represent cDNA amplified by common *Brwd1* primers. Lane 1 = wildtype, Lane 2 = *Brwd1*$^{FlagHA/+}$, Lane 3 = *Brwd1*$^{FlagHA/FlagHA}$ and Lane 4 = water control. Lanes 5-8 represent cDNA amplified by epitope-specific primers. Lane 1 = wildtype, Lane 2 = *Brwd1*$^{FlagHA/+}$, Lane 3 = *Brwd1*$^{FlagHA/FlagHA}$ and Lane 4 = water control. Experiment repeated >3X. **(f)** Western blotting (uncropped) of testis protein probed with anti-HA. Lane 1 and 2 = *Brwd1*$^{FlagHA/+}$, Lane 3 = *Brwd1*$^{FlagHA/FlagHA}$, and Lanes 4 and 5 = wildtype. **(g)** Immunolabeled testis cross-sections from 8-week old males. DDX4 labels cytoplasm, HA labels epitope-tagged BRWD1, and hematoxylin counterstains the nucleus. BRWD1, as expected, is localized in nuclei of spermatocytes. Experiment repeated >3X. Data are presented as averages ± SEM. Source data are provided as a source data file.

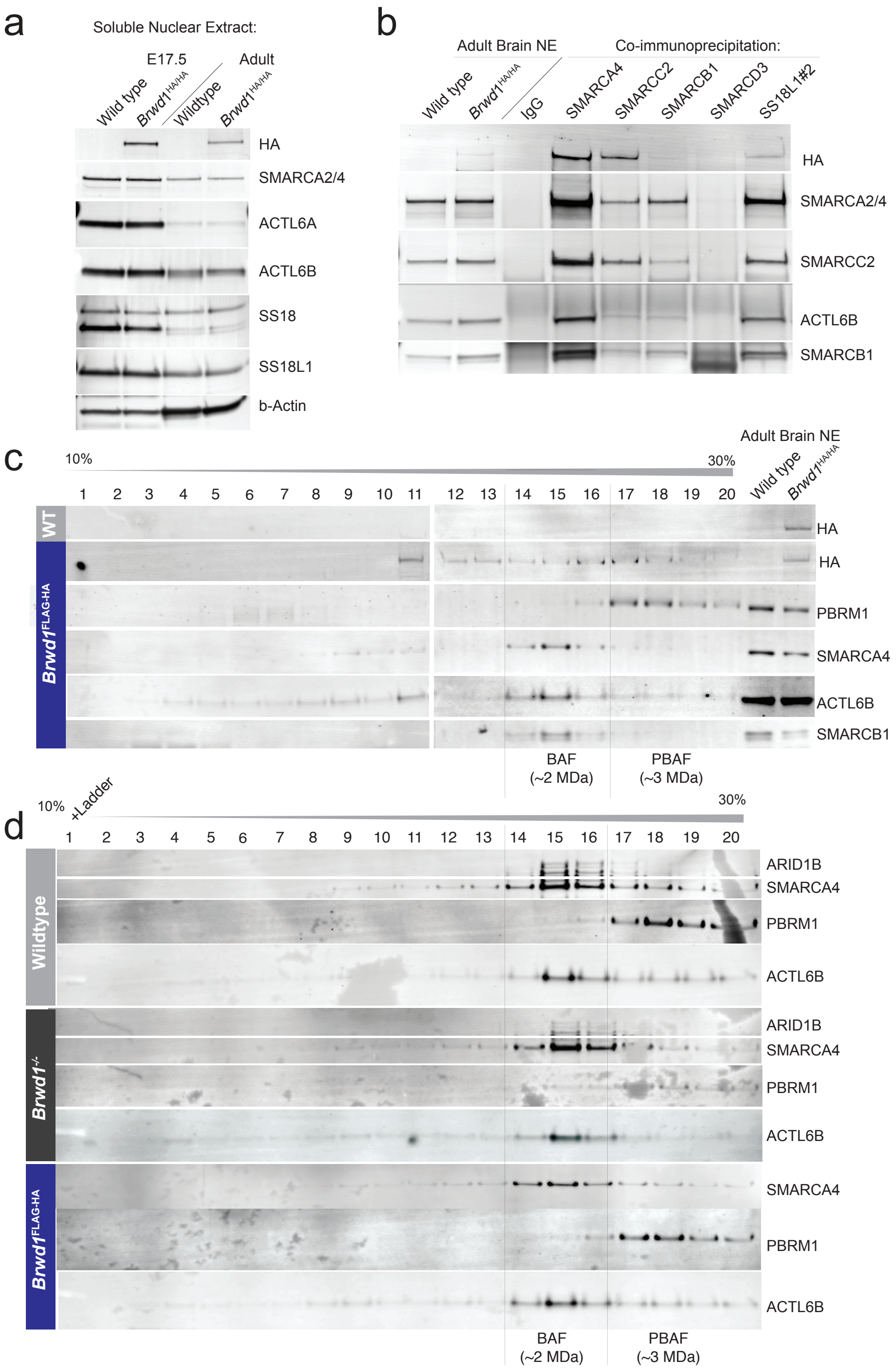

Supplementary Figure 11. BRWD1 migrates at over 2 Mda on glycerol gradients and interacts with BAF complexes in brain nuclear extracts.

(a) Detection of endogenous BRWD1-FLAG-HA protein in embryonic day 17.5 (E17.5) and adult mouse brain soluble nuclear protein extracts (NE) by immunoblotting with an antibody against HA. 7.5 µg of NE was loaded in each lane. **(b)** Glycerol gradient sedimentation analysis of whole brain NE from adult wildtype or *Brwd1*$^{\text{FLAG-HA}}$ mice indicates that BRWD1 is predominantly associates with high molecular weight protein complex(es). Subunits of the BAF complex are used as markers: SMARCA4 and BAF47 peaks indicate the 2 Mda canonical BAF complex and the PBRM1 peak indicates the 3 Mda PBAF complex. BRWD1-FLAG-HA also co-migrates with the neuronal-specific BAF subunit ACTL6B. **(c)** Antibodies to BAF subunits SMARCA4, SMARCC2, SS18L1 (#2, to an internal peptide) and, to some degree, SMARCB1 co-immunoprecipitate endogenously tagged BRWD1-FLAG-HA protein from adult mouse brain nuclear extract. SMARCA4, SMARCC2, and SMARCB1 are constitutive subunits, and SMARCD3 and SS18L1 are neuronal-enriched BAF subunits. The SMARCD3 antibody was ineffective at pulling down BAF. **(d)** Glycerol gradient sedimentation analysis of adult mouse brain NE from *Brwd1*$^{-/-}$ *vs.* wildtype or HA-tagged BRWD1 mice. Genetic deletion of *Brwd1* does not affect the migration of BAF complexes. Experiments repeated >3X. Source data are provided as a source data file. See **Supplemental Figure 15** for uncropped blots with MW markers.

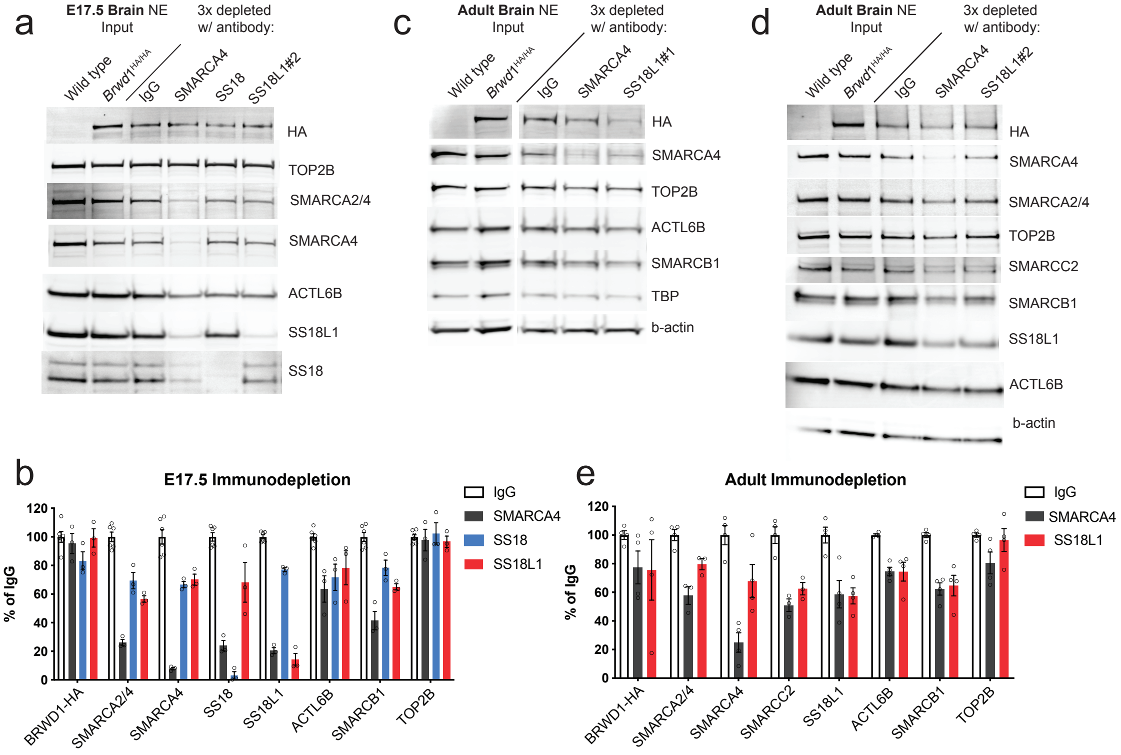

Supplementary Figure 12. Antibodies against the BAF complex partially co-immunodeplete BRWD1 and other BAF subunits from brain nuclear extract.

(a) Embryonic day 17.5 (E17.5) *Brwd1*$^{FLAG\text{-}HA}$ brain soluble nuclear extracts (NE) were subjected to 3 rounds of immunodepletion with antibodies to the BAF ATPase SMARCA4, the neural progenitor BAF subunit SS18, the neuronal BAF subunit SS18L1 (antibody #2), or IgG as a control. Immunoblotting for the C-terminal HA tag of endogenous BRWD1-FLAG-HA revealed modest co-immunodepletion with SS18. BAF subunits that were not direct targets of the antibody were likewise only modestly immunodepleted while subunits that were targeted by the antibody were selectively and almost completely depleted from the NE. **(b)** Quantification of **a** including replicate experiments (n=3) indicates ~18% co-immunodepletion of BRWD1 and ~25% immunodepletion of SMARCA2/4 with SS18 antibody (IgG: n = 6; SMARCA4, SS18 and SS18L1: n = 3). **(c-d)** Adult (male, 6 week old) *Brwd1*$^{FLAG\text{-}HA}$ brain soluble NE was subjected to 3

rounds of immunodepletion with SMARCA4, SS18L1 or IgG antibodies. Immunoblotting with HA antibody revealed modest co-immunodepletion of BRWD1-FLAG-HA and non-target BAF subunits with SMARCA4 antibody. The C-terminal SS18L1#1 antibody in c robustly co-immunodepleted BRWD1-FLAG-HA (n=1 experiment) while the internal SS18L1#2 antibody in d only slightly reduced BRWD1-FLAG-HA signal in the NE (representative of n=3 experiments). **(e)** Quantification of all 4 experiments represented by c-d indicates that BRWD1-FLAG-HA is co-immunodepleted by ~20% on average with antibodies to BAF [IgG: n = 4 for all proteins; SMARCA4: n = 4 for all proteins except SMARCA2/4 and SS18L1 (n = 3)]. Data are presented as averages ± SEM. Source data are provided as a source data file. See **Supplemental Figure 15** for uncropped blots with MW markers.

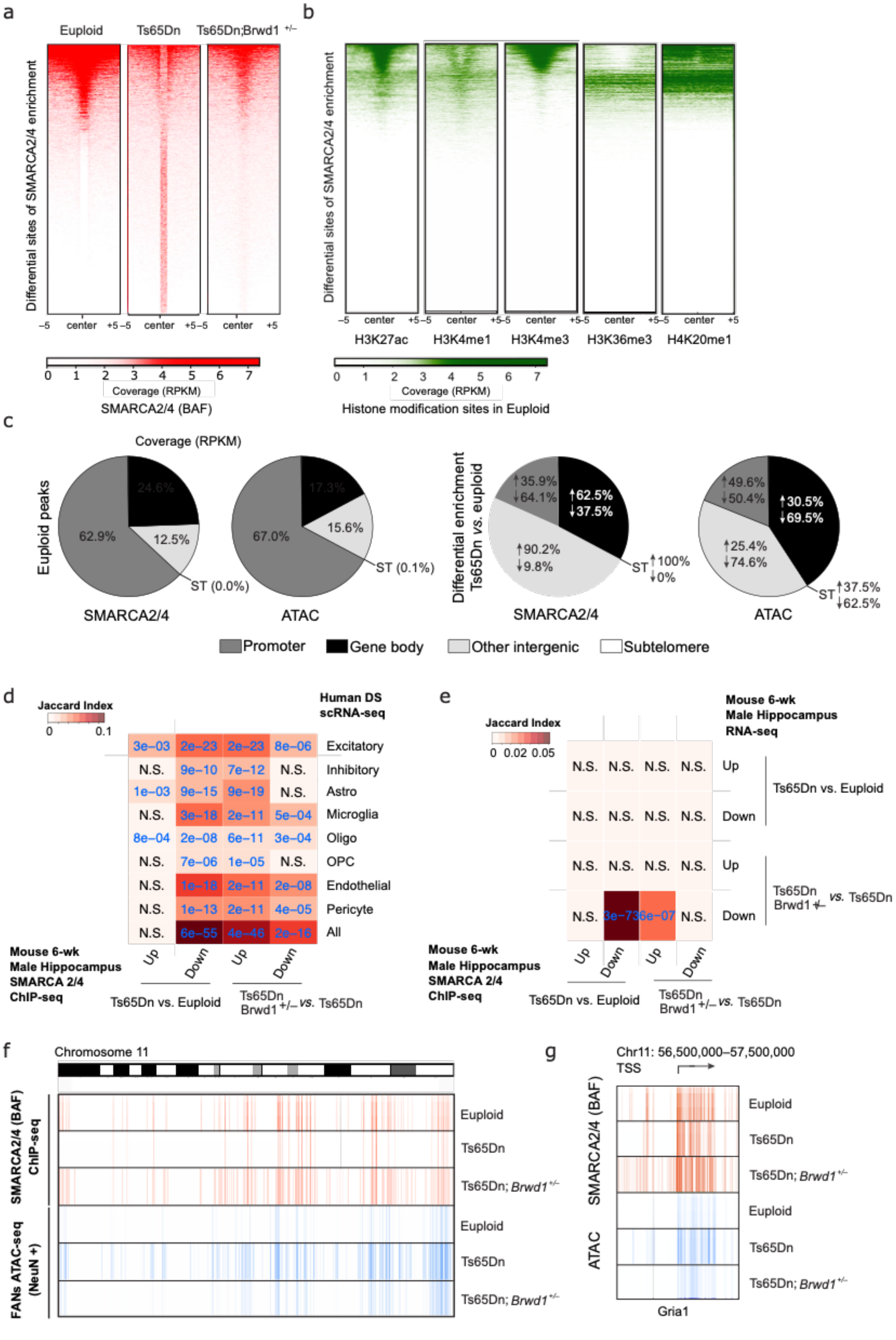
a
Euploid
Ts65Dn
Ts65Dn;Brwd1 +/−
Differential sites of SMARCA2/4 enrichment
Coverage (RPKM)
SMARCA2/4 (BAF)
b
Differential sites of SMARCA2/4 enrichment
H3K27ac
H3K4me1
H3K4me3
H3K36me3
H4K20me1
Coverage (RPKM)
Histone modification sites in Euploid
c
Coverage (RPKM)
Euploid peaks
SMARCA2/4
ATAC
Differential enrichment Ts65Dn vs. euploid
Promoter
Gene body
Other intergenic
Subtelomere
d
Jaccard Index
Human DS scRNA-seq
Excitatory
Inhibitory
Astro
Microglia
Oligo
OPC
Endothelial
Pericyte
All
Mouse 6-wk Male Hippocampus SMARCA 2/4 ChIP-seq
Ts65Dn vs. Euploid
Ts65Dn Brwd1 +/− vs. Ts65Dn
e
Jaccard Index
Mouse 6-wk Male Hippocampus RNA-seq
Ts65Dn vs. Euploid
Ts65Dn Brwd1 +/− vs. Ts65Dn
Mouse 6-wk Male Hippocampus SMARCA 2/4 ChIP-seq
f
Chromosome 11
SMARCA2/4 (BAF) ChIP-seq
FANs ATAC-seq (NeuN +)
Euploid
Ts65Dn
Ts65Dn; Brwd1 +/-
g
Chr11: 56,500,000–57,500,000
TSS
SMARCA2/4 (BAF)
ATAC
Gria1

Supplementary Figure 13. SMARCA2/4 is genically enriched in euploid hippocampus and displays alterations in genomic distribution in trisomic brain.

(a) Genomic distribution of (left) SMARCA2/4 and ATAC peaks in euploid adult male hippocampus, as determined via MACSv2.1.124-based peak calling (FDR<0.05, FC>1.2 cutoffs applied after adjusting for multiple comparisons; n = 4/group; normalized against respective DNA inputs). **(b)** Using these same genomic coordinates, normalized enrichment heatmaps for permissive (H3K27ac, H3K4me1, H3K4me3, H3K36me3 and H4K20me1) histone modifications are provided. **(c)** diffReps analysis of (left) SMARCA2/4 and (right) ATAC differential enrichment comparing Ts65Dn *vs.* euploid adult male hippocampus (p<0.0001 cutoff applied; n = 3-4/group). Pie charts indicate distribution of differential enrichment events. **(d)** Odds ratio analyses (Fisher's exact test) depicting adj. p-values for overlap between PCGs displaying differential SMARCA2/4 enrichment and human DS RNA-seq profiles. **(e)** Odds ratio analyses (Fisher's exact test) depicting adj. p-values for overlap between PCGs displaying differential SMARCA2/4 enrichment and adult male Ts65Dn RNA-seq profiles. Representative genome browser tracks (igv, displayed in heatmap form) of **(f)** chromosome 11 (left) and **(g)** the *Gria1* gene locus for Smarca2/4 and chromatin accessibility (ATAC) in euploid *vs.* Ts65Dn *vs.* Ts65Dn;*Brwd1*$^{+/-}$ adult male hippocampus. Source data are provided as a source data file.

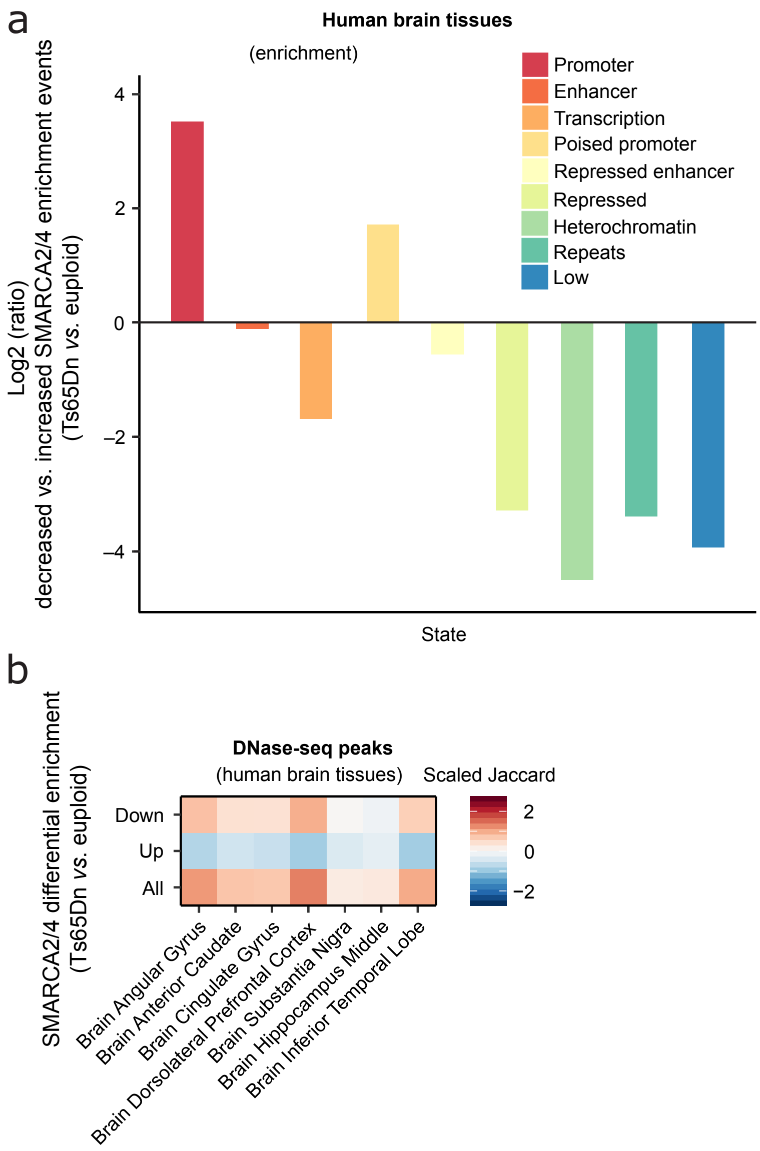

Supplementary Figure 14. Mapping SMARCA2/4 differential enrichment events in trisomic mouse hippocampus against chromatin states identified in human brain.

(**a**) Comparison of log2 ratio of relative frequency between sites with significantly decreased *vs.* increased enrichment per chromatin state. (**b**) Overlap between open chromatin of seven brain regions assayed by DNase-seq from the Roadmap Epigenomics Project and significant differentially enriched sites for SMARCA2/4 (Ts65Dn *vs.* euploid). The overlap was quantified

using the “scaled” Jaccard index obtained by calculating standard deviations after subtracting the mean Jaccard index of the sample.
Up - sites with significantly increased SMARCA2/4 enrichment events; Down – sites with significantly decreased enrichment events; All: sites significantly increased or decreased enrichment events. Source data are provided as a source data file.

Supplemental Figure 15. Uncropped western blots with MW markers

Raw Western Blot scans for Fig. 3B:

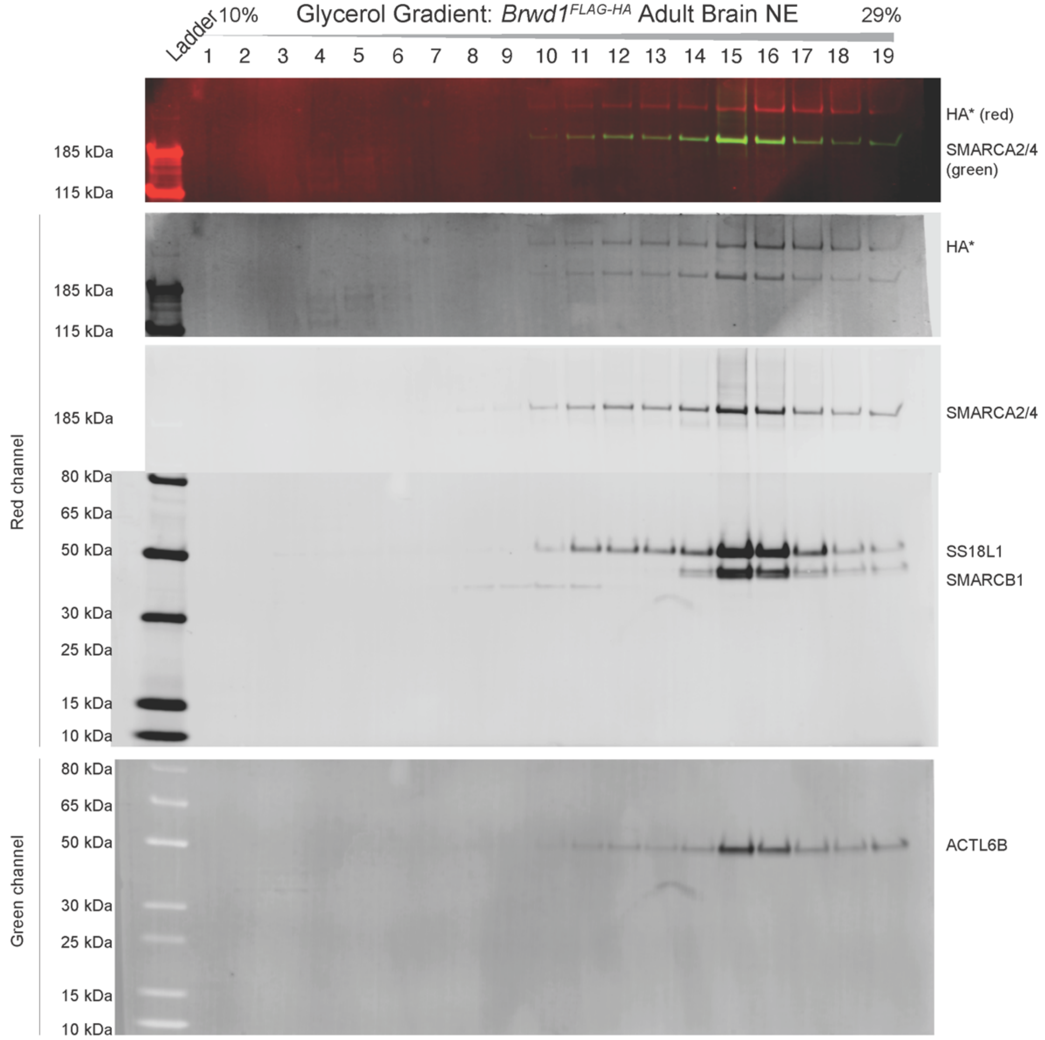

*BRWD1-FLAG-HA is approximately 260 kDA

Raw Western Blot scans for Fig. 3C:

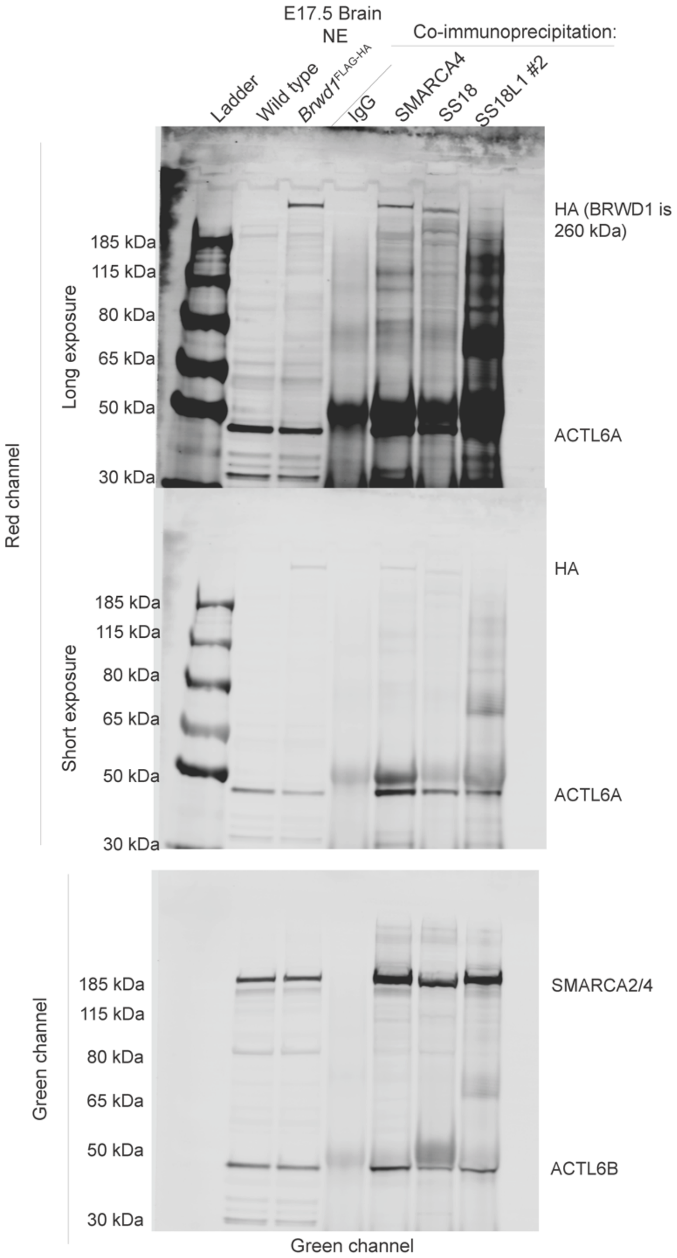

Raw Western Blot scans for Fig. 3D:

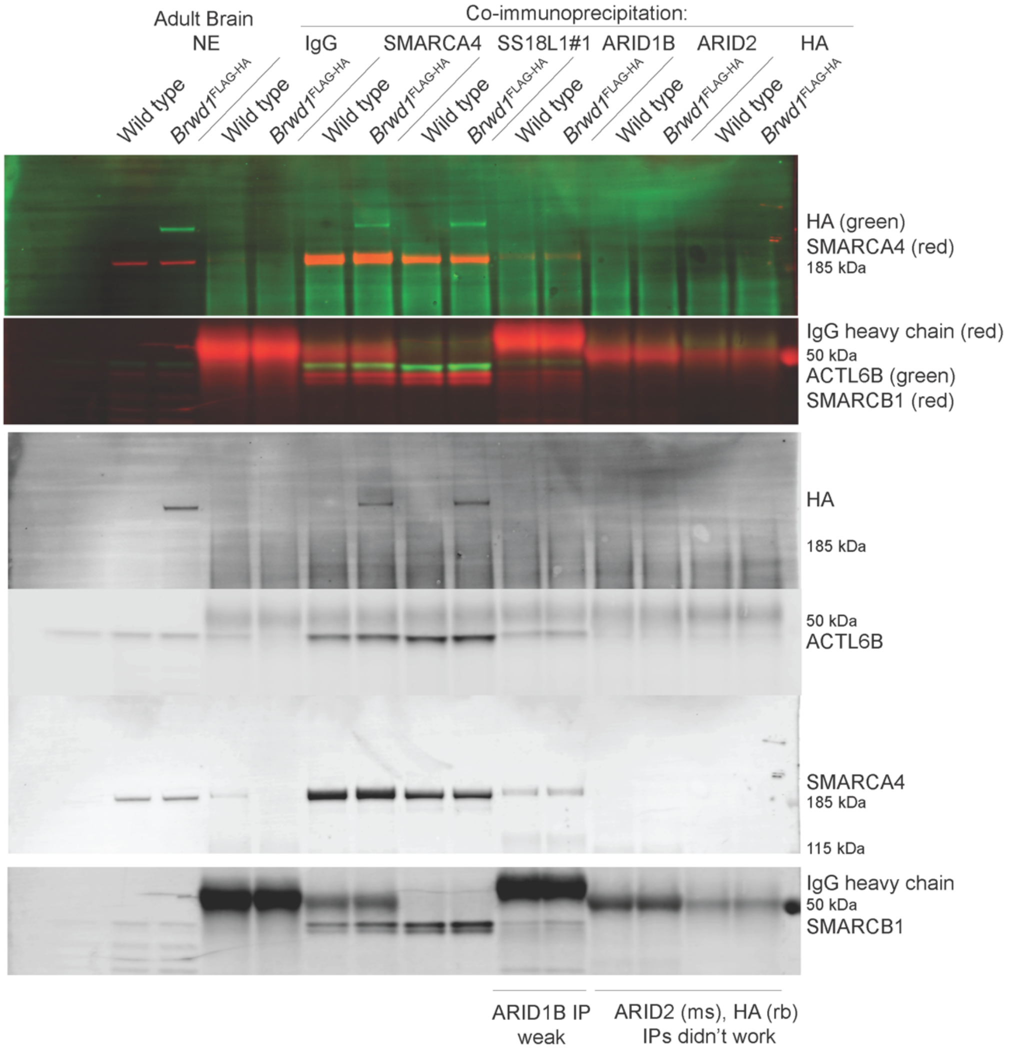

Raw Western Blot scans for Fig. 3E:

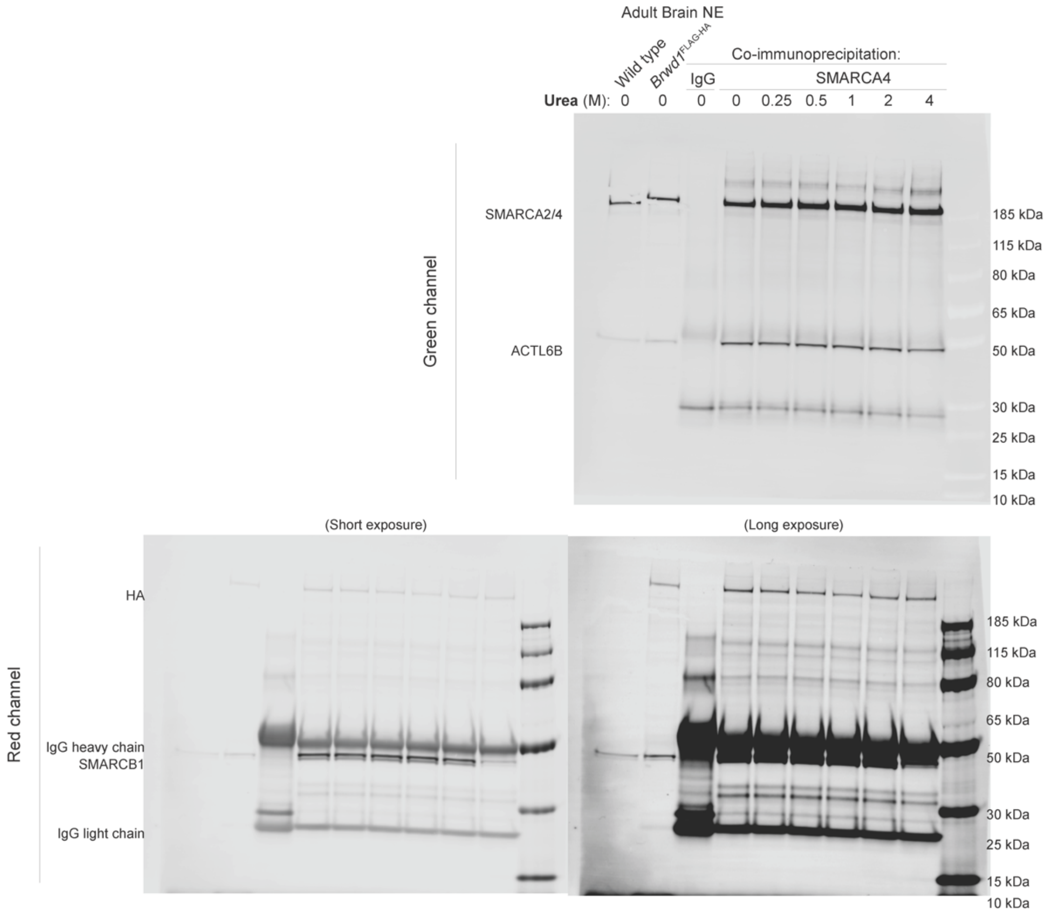

Replicate urea denaturation data used to generate Fig. 3F

Urea (M)	Brwd1-HA			Baf53b			Baf47		
X	A:Y1	A:Y2	A:Y3	B:Y1	B:Y2	B:Y3	C:Y1	C:Y2	C:Y3
0.00	100.000000000	100.000000000	100.000000000	99.137531860	100.000000000	87.079934180	86.614148220	100.000000000	91.747532160
0.25	62.471172420	66.980000000	59.879847280	100.000000000	70.850000000	100.387556600	100.000000000	81.290000000	115.680708100
0.50	36.904542390	44.120000000	25.572633770	88.615989590	68.590000000	93.821982920	97.540701930	80.320000000	98.902218480
1.00	42.007932760	29.950000000		87.582770950	67.810000000		98.361889500	77.990000000	
2.00	36.214648180	42.020000000	54.793494950	82.428897140	69.830000000	118.710526300	74.902697100	65.270000000	81.460083380
4.00		36.740000000	43.757618740		49.090000000	73.411251410		8.620000000	4.550811323

Raw Western Blot scans for Extended Data Fig. 11A:

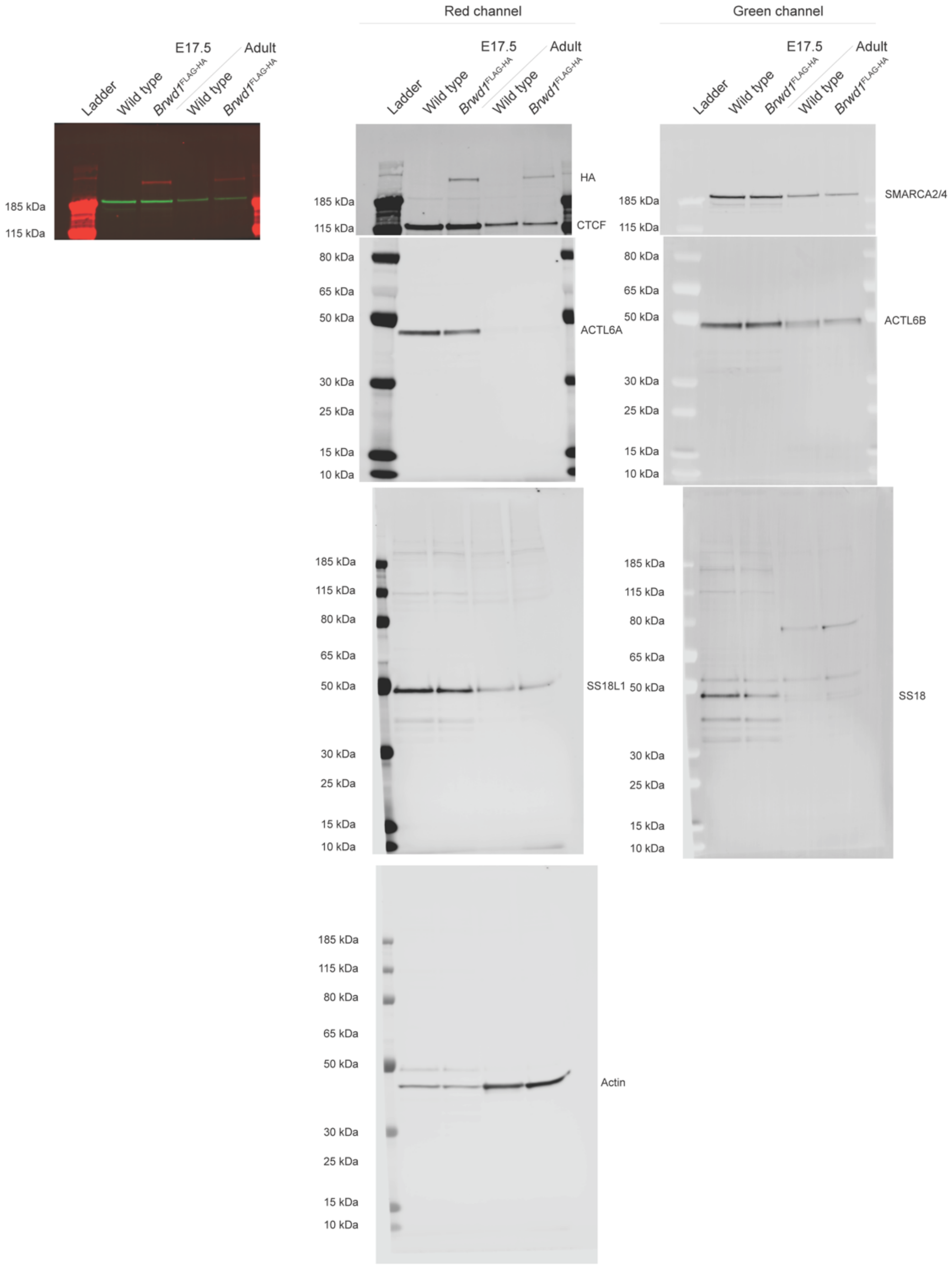

Raw Western Blot scans for Extended Data Fig. 11B:

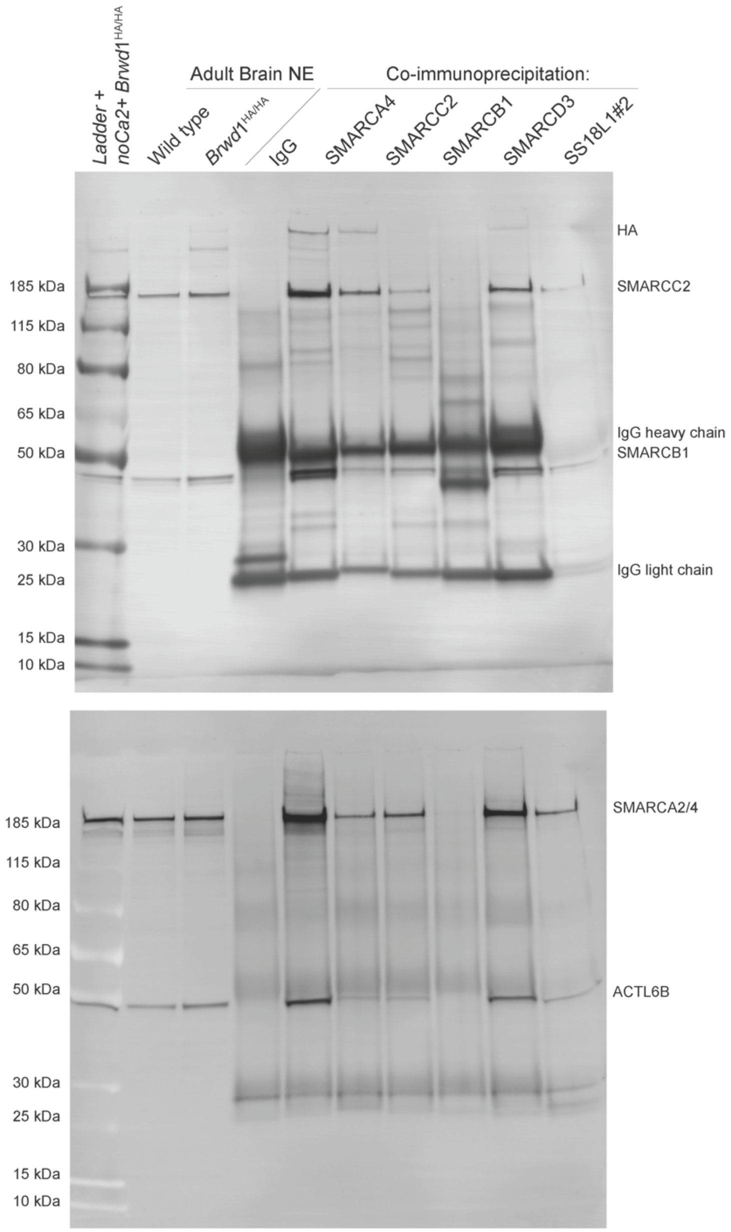

Red channel: HA (BRWD1~260 kDA), SMARCC2 (170 kDA), SMARCB1 (44 kDA)
Green chnnel: SMARCA2/4 (180 kDA), ACTL6B (47 kDa)

Raw Western Blot scans for Extended Data Fig. 11C:

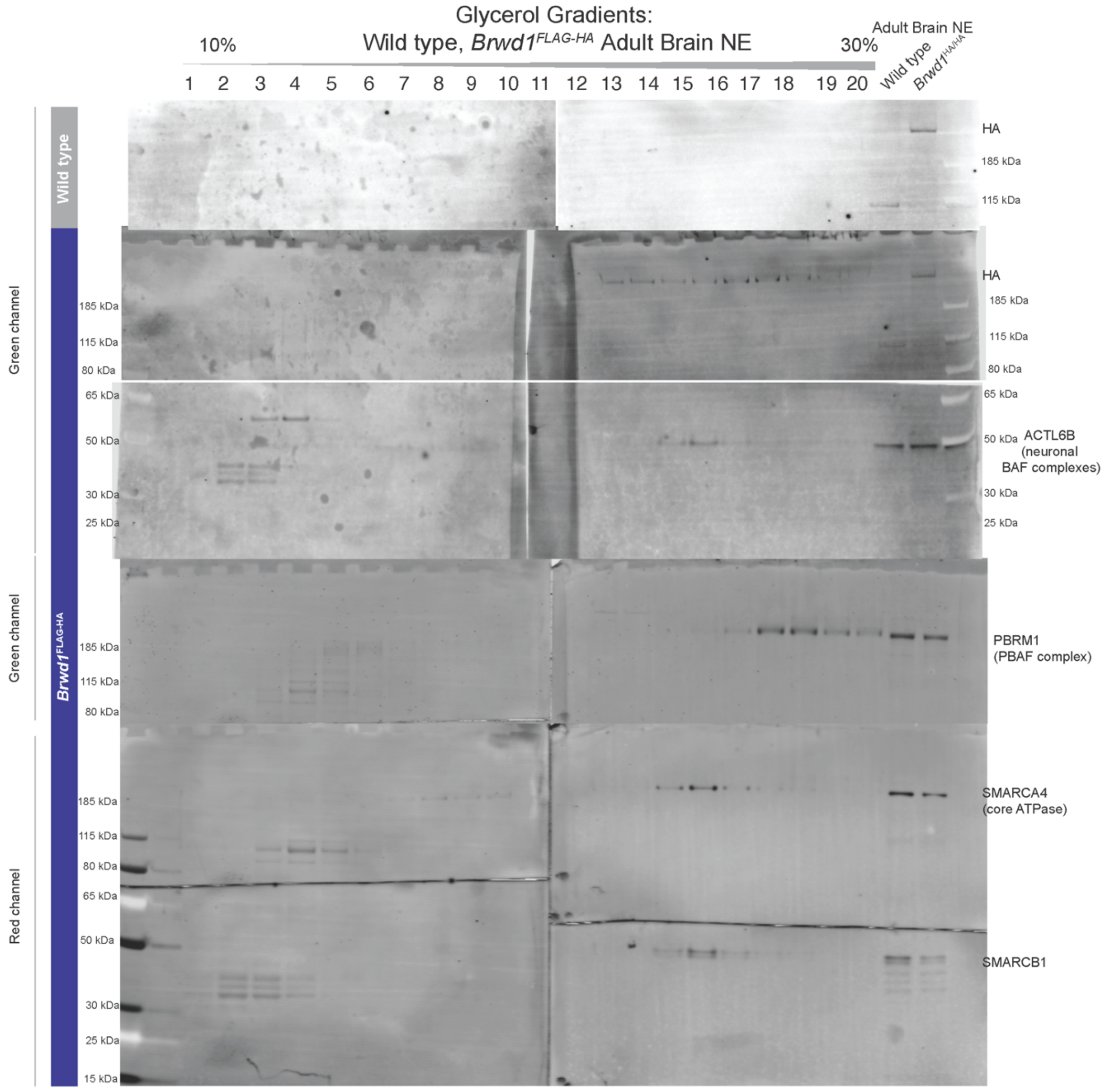

Raw Western Blot scans for Extended Data Fig. 11D, part 1:

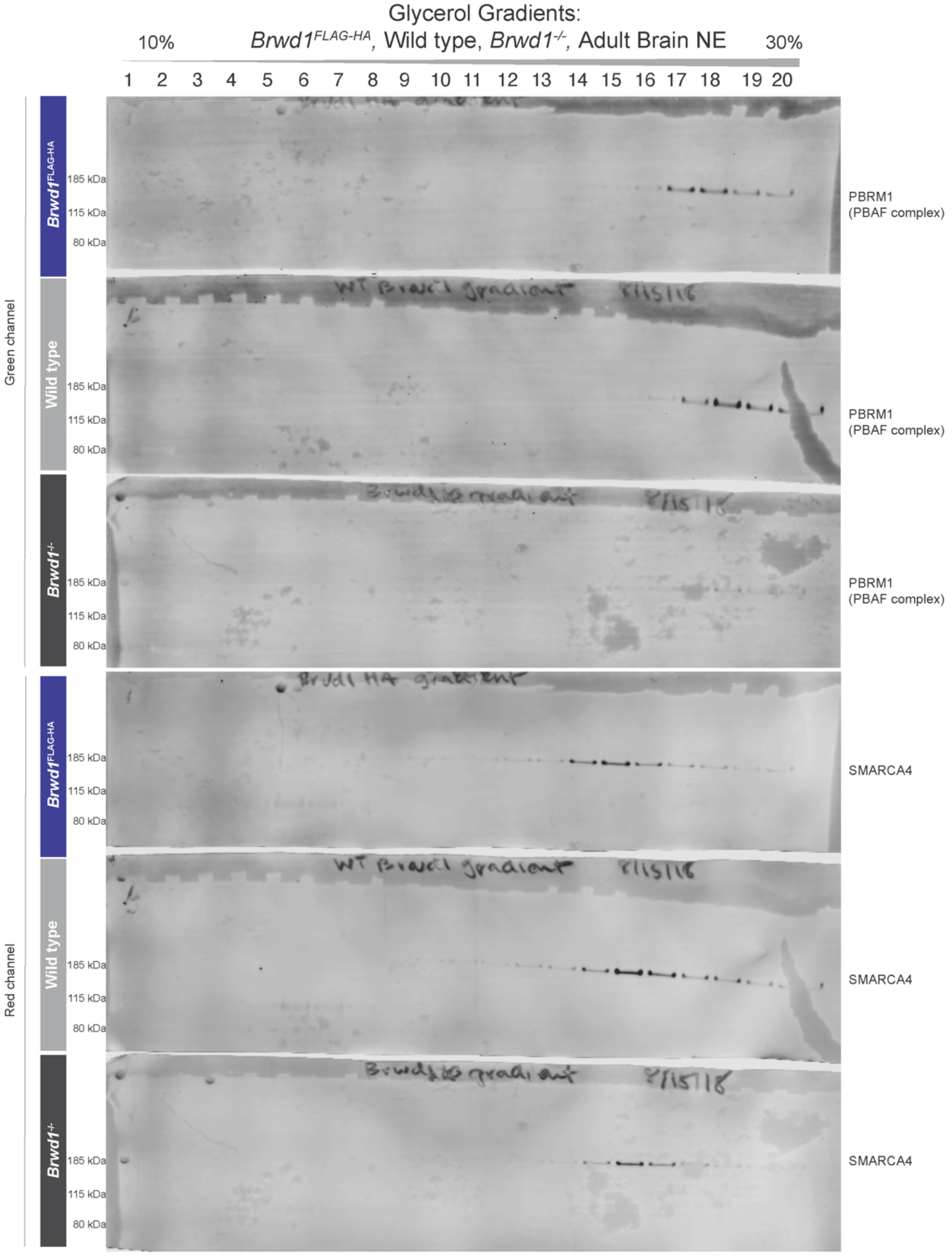

Raw Western Blot scans for Extended Data Fig. 11D, part 2:

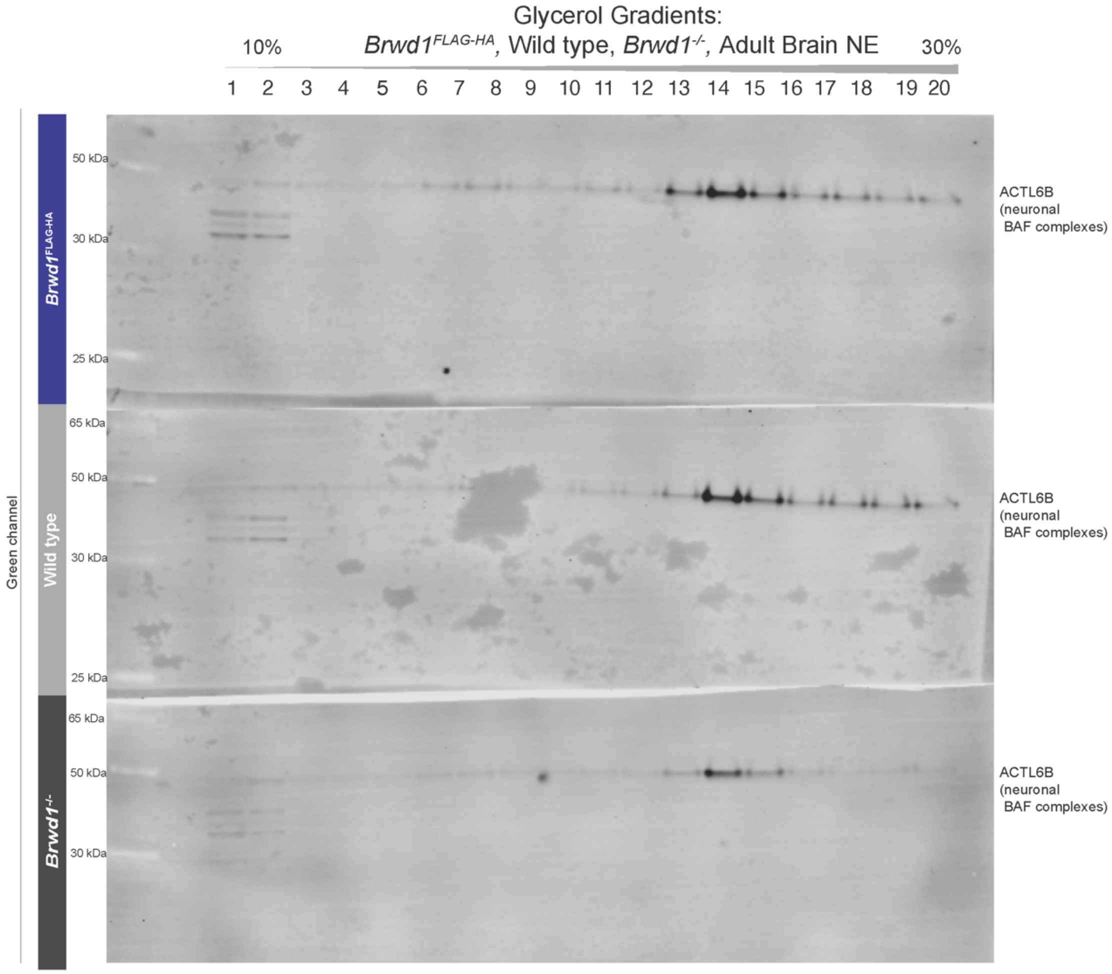

Raw Western Blot scans for Extended Data Fig. 11D, part 3:

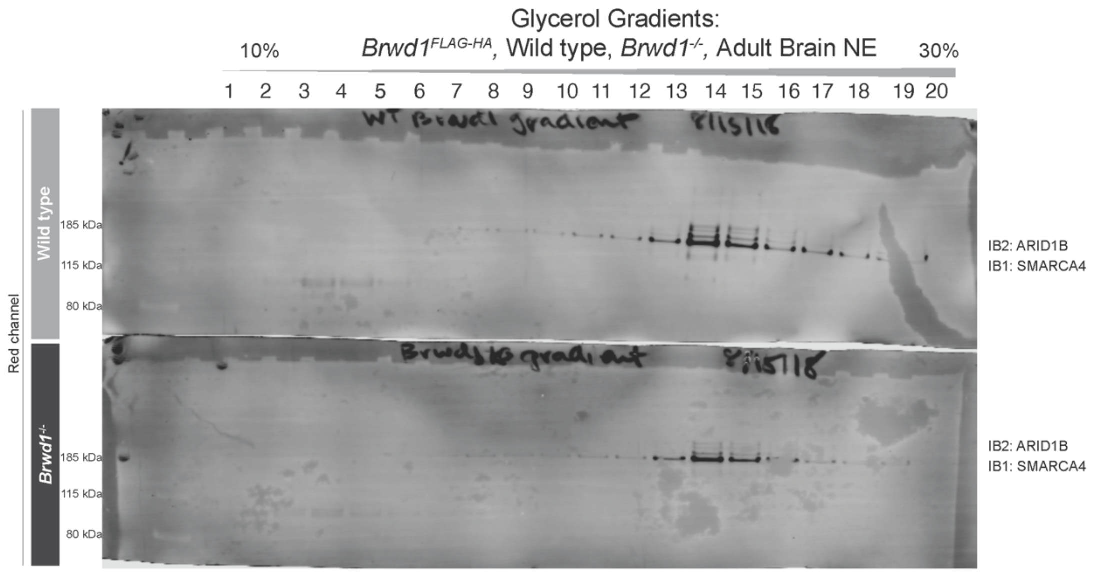

Raw Western Blot scans for Extended Data Fig. 12A

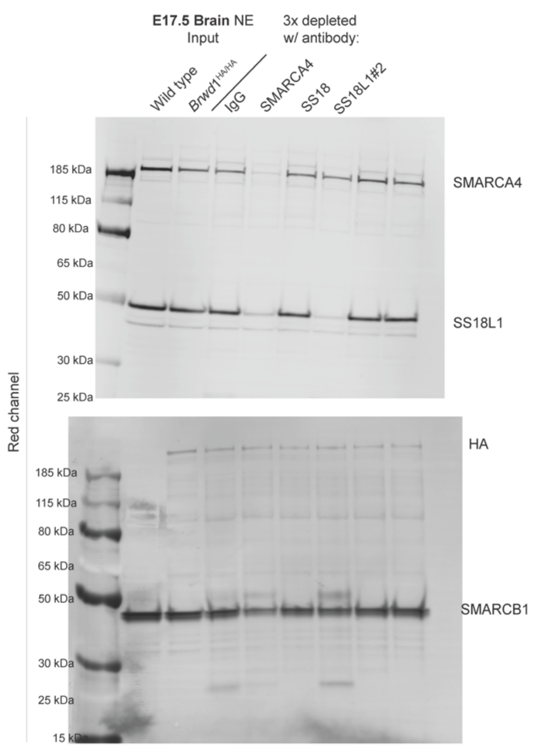

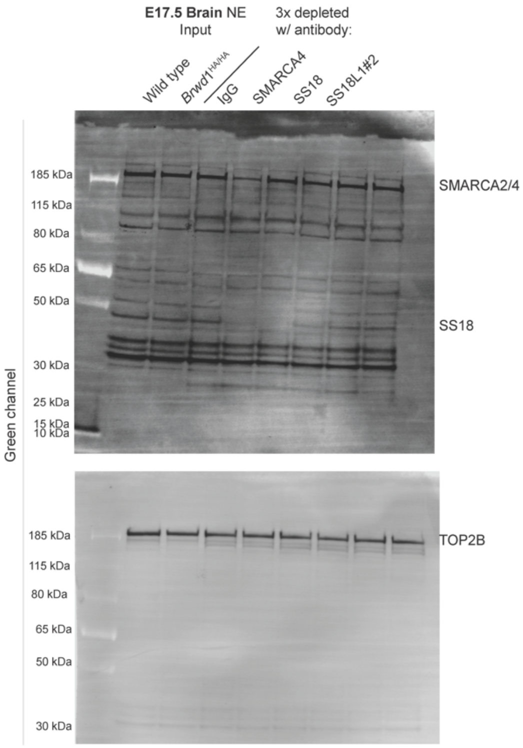

Replicate E17 Immunodepletion data shown in Extended Data Fig. 12B

		IgG						SMARCA4			SS18			SS18L1		
		A:Y1	A:Y2	A:Y3	A:Y4	A:Y5	A:Y6	B:Y1	B:Y2	B:Y3	C:Y1	C:Y2	C:Y3	D:Y1	D:Y2	D:Y3
1	BRWD1-HA	114.46534820	85.53465176	101.91691780	98.08308224	100.77379820	99.22620185	96.105268800	107.170591000	82.874869800	75.140424040	78.873397570	95.616940010	110.497355000	88.245102380	98.976007170
2	SMARCA2/4	106.79050170	93.20949833	105.92166920	94.07833080	90.58689252	109.41310750	24.229663240	30.501950680	23.670738250	66.881706350	61.024333590	80.307354820	60.958465670	55.477724210	53.350058360
3	SMARCA4	85.71586253	114.28413750	105.43611650	94.56388352	87.55134241	112.44865760	6.224380441	9.237941498	8.225159079	63.375615620	70.865941380	65.683393980	67.159035110	77.691855940	65.361372120
4	SS18	107.60410130	92.39589871	106.55646150	93.44353846	105.49908640	94.50091364	20.742021860	31.183359020	19.915712470	0.235602321	8.375144703	0.958939734	64.722652280	93.915370650	46.072144940
5	SS18L1	96.63841768	103.36158230	96.28312386	103.71687610	97.25610257	102.74389740	17.341757350	24.500649890	19.591174200	77.930192770	74.422378300	78.973530060	9.501439596	22.941782210	9.970149302
6	ACTL6B	91.92852768	108.07147230	100.89357050	99.10642947	103.01268130	96.98731867	45.622827910	75.932344010	69.056122320	53.967200240	84.375883930	77.043823340	54.761498490	88.013469380	92.260256340
7	SMARCB1	90.97563143	109.02436860	103.82657640	96.17342360	92.46619536	107.53380460	54.313221590	35.915857570	34.141762470	88.095576050	77.622931590	69.573885600	62.118692160	69.336315820	63.566569670
8	TOP2B	94.73012866	105.26987130	95.36597595	104.63402400	98.55007580	101.44992420	84.020206940	98.827407120	110.379780800	95.030648370	94.896855390	117.266443700	90.572453830	103.957576100	95.290566990

Raw Western Blot scans for Extended Data Fig. 12C

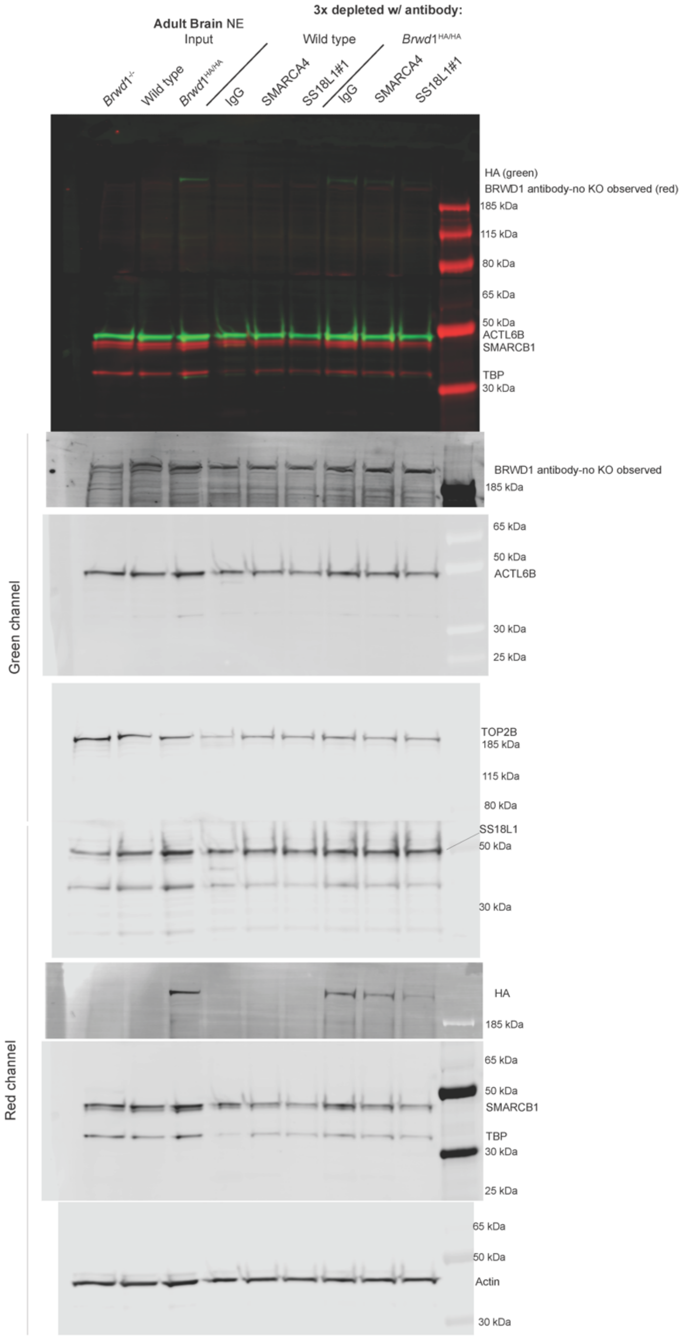

Raw Western Blot scans for Extended Data Fig. 12D

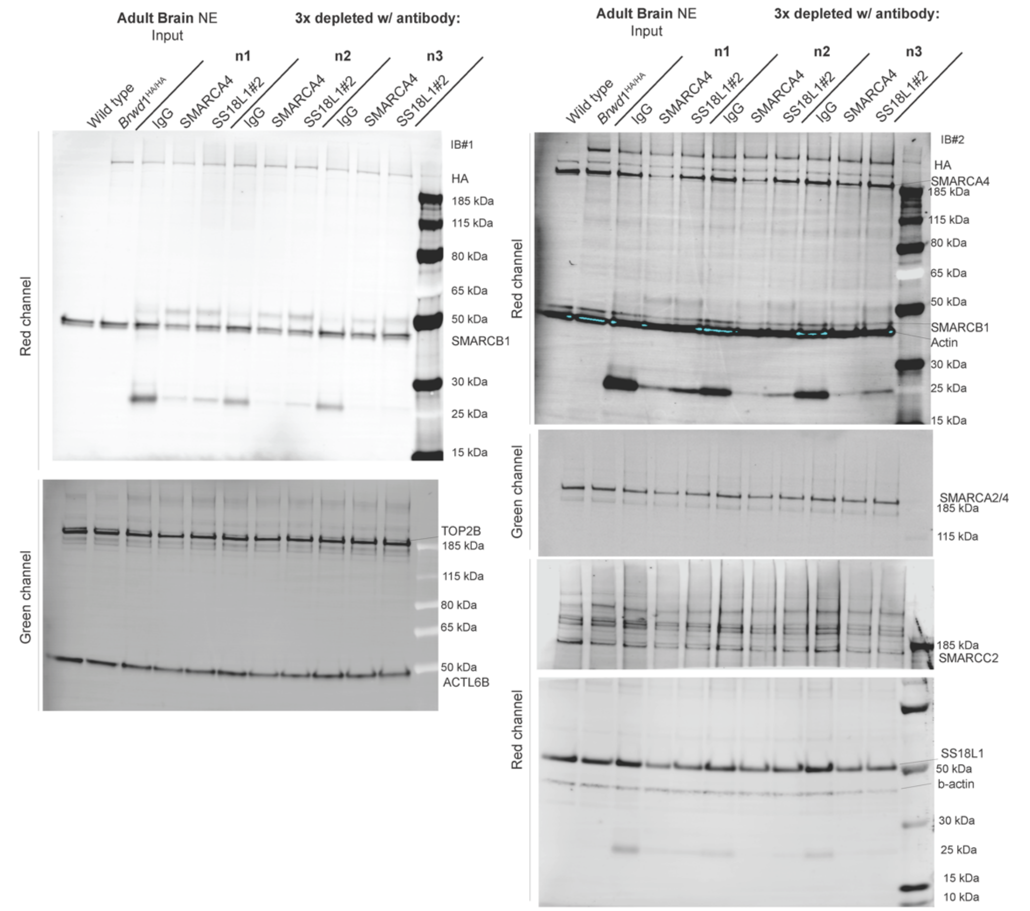

Replicate Adult Immunodepletion data shown in Extended Data Fig. 12E

	IgG				SMARCA4				SS18L1			
	A:Y1	A:Y2	A:Y3	A:Y4	B:Y1	B:Y2	B:Y3	B:Y4	C:Y1	C:Y2	C:Y3	C:Y4
BRWD1-HA	91.938617770	101.465845900	106.595536300	100.000000000	56.004556940	94.753016590	99.611719620	59.080000000	72.313252890	101.706410100	111.045805900	17.530000000
SMARCA2/4	88.998178600	105.397313300	105.604508100	100.000000000	45.602327000	64.424934000	63.386664800		71.839602600	83.885549100	83.548314600	
SMARCA4	81.132954900	106.912257600	111.954787500	100.000000000	8.429931300	19.365107400	33.514611500	38.520000000	56.120092400	68.288260500	99.984665000	47.000000000
SMARCC2	83.998766600	106.131030000	109.870203400	100.000000000	42.815790900	52.366580200	57.684651600		62.060444700	70.263475300	55.230074100	
SS18L1	103.117172500	85.127596900	111.755230600	100.000000000	38.879702700	59.321240300	51.948888800	84.390000000	56.248478500	70.670108600	59.352378100	43.520000000
ACTL6B	98.424957900	99.288780500	102.286261500	100.000000000	72.230269600	67.980068200	82.295160300	75.690000000	81.417557700	78.265638900	83.121936900	54.960000000
SMARCB1	95.272182260	100.963323200	103.764494600	100.000000000	50.295156540	62.144324190	68.886682610	67.700000000	68.597347690	67.777166450	78.294693710	44.240000000
TOP2B	96.097728600	100.731106900	103.171164600	100.000000000	65.203429400	89.226629500	97.394366500	70.240000000	99.079815500	96.536109200	114.725920100	75.770000000
