# Supplementary material for: Rescue of deficits by Brwd1 copy number restoration in the Ts65Dn mouse model of Down syndrome
Source: Nat Commun. 2022 Oct 26;13:6384. doi: 10.1038/s41467-022-34200-0 (PMC9606253; doi:10.1038/s41467-022-34200-0)
Supplement: Supplementary file 13 — Reporting Summary [file 41467_2022_34200_MOESM13_ESM.pdf]

nature portfolio

Corresponding author(s): Ian Maze, PhD

Last updated by author(s): Oct 5, 2022

Reporting Summary

Nature Portfolio wishes to improve the reproducibility of the work that we publish. This form provides structure for consistency and transparency in reporting. For further information on Nature Portfolio policies, see our Editorial Policies and the Editorial Policy Checklist.

Statistics

For all statistical analyses, confirm that the following items are present in the figure legend, table legend, main text, or Methods section.

n/a	Confirmed	
☐	☒	The exact sample size (*n*) for each experimental group/condition, given as a discrete number and unit of measurement
☐	☒	A statement on whether measurements were taken from distinct samples or whether the same sample was measured repeatedly
☐	☒	The statistical test(s) used AND whether they are one- or two-sided<br>*Only common tests should be described solely by name; describe more complex techniques in the Methods section.*
☒	☐	A description of all covariates tested
☐	☒	A description of any assumptions or corrections, such as tests of normality and adjustment for multiple comparisons
☐	☒	A full description of the statistical parameters including central tendency (e.g. means) or other basic estimates (e.g. regression coefficient) AND variation (e.g. standard deviation) or associated estimates of uncertainty (e.g. confidence intervals)
☐	☒	For null hypothesis testing, the test statistic (e.g. *F*, *t*, *r*) with confidence intervals, effect sizes, degrees of freedom and *P* value noted<br>*Give P values as exact values whenever suitable.*
☒	☐	For Bayesian analysis, information on the choice of priors and Markov chain Monte Carlo settings
☒	☐	For hierarchical and complex designs, identification of the appropriate level for tests and full reporting of outcomes
☒	☐	Estimates of effect sizes (e.g. Cohen's *d*, Pearson's *r*), indicating how they were calculated

Our web collection on statistics for biologists contains articles on many of the points above.

Software and code

Policy information about availability of computer code

Data collection	No custom algorithms were used.
Data analysis	RNA-SEQ ANALYSES<br>Raw sequencing reads from mouse embryonic forebrain, e16 primary neuronal cultures, or adult hippocampus were mapped to mm10 using HISAT2(v2.2.1+galaxy0). Counts of reads mapping to genes were obtained using featureCounts(v2.0.1+galaxy2) against Ensembl v90 annotation. Read counts were normalized using RUVr (v1.24.0), and differential expression analysis was done using the DESeq2 package (v1.6.3) – for both likelihood-ratio-test and pairwise comparisons – at FDR cutoffs of 0.1. GO analyses were conducted using iDEP. Odds ratio analyses between DE gene lists and human DS RNA-seq were conducted based on gene names using geneOverlap (GeneOverlap: Test and visualize gene overlaps. R package v1.23.0.<br><br>CHIP-SEQ/ATAC-SEQ ANALYSES<br>For SMARCA2/4 ChIP-seq and neuronal ATAC-seq from adult mouse hippocampus, raw sequencing reads were aligned to the mouse genome (mm10) using default settings of HISAT2. Only uniquely mapped reads were retained. Alignments were filtered using SAMtools (v1.19) to remove duplicate reads. For ChIP-seq, peak-calling–normalized to respective inputs–was performed using MACS (v2.1.124) with default settings; the window size was set as 300 bp. For ATAC-seq, peak calling was performed using MACS (v2.1.124) with settings --nomodel --shift -100 --extsize 200. For both ChIP-seq and ATAC-seq datasets, peaks were filtered for FDR < 0.05 and fold change > 1.2. Differential analyses were performed using diffReps with a window size of 1 kb. A default p-value cutoff of 0.0001 was used. Peaks and differential sites were further annotated to nearby genes or intergenic regions using the region analysis tool from the diffReps package (v1.55.6). Histone PTM enrichment data in mouse hippocampus were extracted from published sources. ATAC-seq differential lists were compared to ChIP-seq differential lists based on gene names using geneOverlap (GeneOverlap: Test and visualize gene overlaps. R package version 1.23.0, http://shenlab-sinai.github.io/shenlab-sinai/), and heat maps were drawn using the deepTools package.

Overlap with existing epigenomic annotations
We computed the overlap of significant differentially bound sites of SMARCA2/4 (Ts65Dn vs. euploid) and chromatin states from the Epigenomics Roadmap Project using the scaled Jaccard index, obtained by calculating standard deviations after subtracting the mean of the sample (Jaccard index is an intersection of base pairs divided by the union of base pairs). We used the "expanded" chromHMM 18-state (6 histone marks, 98 epigenome model) for seven brain regions, i.e., angular gyrus, anterior caudate, cingulate gyrus, dorsolateral prefrontal cortex, hippocampus, inferior temporal lobe and substantia nigra. To improve interpretability, we consolidated the 18 states into 9 states as follows: Promoter (TssA, TssFlnk, TssFlnkU, and TssFlnkD), Enhancer (EnhG1, EnhG2, EnhA1, EnhA2, EnhWk), Transcription (Tx, TxWk), Poised promoter (TssBiv), Repressed enhancer (EnhBiv), Repressed (ReprPC, ReprPCWk), Heterochromatin (Het), Repeats (ZNF/Rpts) and Low (Quies).
To compare our significant differentially bound sites with known regions of open chromatin in different brain regions, we used imputed versions of DNase-seq datasets from Epigenomics Roadmap Project.

PATCH ELECTROPHYSIOLOGY
sEPSC and sIPSC were analyzed with the MiniAnalysis 6.0.3 software (Synaptosoft)

For manuscripts utilizing custom algorithms or software that are central to the research but not yet described in published literature, software must be made available to editors and reviewers. We strongly encourage code deposition in a community repository (e.g. GitHub). See the Nature Portfolio guidelines for submitting code & software for further information.

Data

Policy information about availability of data

All manuscripts must include a data availability statement. This statement should provide the following information, where applicable:
- Accession codes, unique identifiers, or web links for publicly available datasets
- A description of any restrictions on data availability
- For clinical datasets or third party data, please ensure that the statement adheres to our policy

Data and Materials Availability: Data from RNA-seq, ChIP-seq and ATAC-seq experiments have been deposited in the National Center for Biotechnology Information Gene Expression Omnibus (GEO) database under accession numbers GSE210117 and GSE151255. We declare that the data supporting findings for this study are available within the article and Supplementary Information. No restrictions on data availability apply.

Human research participants

Policy information about studies involving human research participants and Sex and Gender in Research.

Reporting on sex and gender	N/A
Population characteristics	N/A
Recruitment	N/A
Ethics oversight	N/A

Note that full information on the approval of the study protocol must also be provided in the manuscript.

Field-specific reporting

Please select the one below that is the best fit for your research. If you are not sure, read the appropriate sections before making your selection.

[x] Life sciences   [ ] Behavioural & social sciences   [ ] Ecological, evolutionary & environmental sciences

For a reference copy of the document with all sections, see nature.com/documents/nr-reporting-summary-flat.pdf

Life sciences study design

All studies must disclose on these points even when the disclosure is negative.

Sample size	Adequate sample sizes are generally determined based upon inter-sample variability. Throughout the manuscript, we determined the significance of results based upon a general confidence interval of 95%. We do not include specific justifications of sample size within the methods (e.g., power analyses), as sample sizes were based on extensive laboratory experience with these endpoints. The sample sizes chosen are consistent with those used by others in the field to achieve statistically significant results comparing euploid vs. Ts65Dn animals.
Data exclusions	No data were excluded.
Replication	All biological endpoints were reliably reproduced using numerous biological (>3 for all experiments in which statistics were employed) and technical replicates for each experiment. All novel tools/mouse lines used in this study were extensively validated, as demonstrated in the

manuscript submission.

Randomization	For all behavioral, electrophysiological and genomic analyses, animals were randomly assigned to groups (segregated by viral treatments and/or genotype). Tissue samples were not pooled from multiple animals in these studies (i.e., each n represents a discrete data point).
Blinding	For all behavioral, electrophysiological and genomic analyses, investigators were blinded to conditions such as genotype, viral treatment conditions, etc. prior to analysis.

Reporting for specific materials, systems and methods

We require information from authors about some types of materials, experimental systems and methods used in many studies. Here, indicate whether each material, system or method listed is relevant to your study. If you are not sure if a list item applies to your research, read the appropriate section before selecting a response.

Materials & experimental systems

n/a	Involved in the study
☐	☒ Antibodies
☒	☐ Eukaryotic cell lines
☒	☐ Palaeontology and archaeology
☐	☒ Animals and other organisms
☒	☐ Clinical data
☒	☐ Dual use research of concern

Methods

n/a	Involved in the study
☐	☒ ChIP-seq
☒	☐ Flow cytometry
☒	☐ MRI-based neuroimaging

Antibodies

Antibodies used

IMMUNOCYTOCHEMISTRY: goat anti-NANOG (R&D AF1997), 1:200; mouse anti-TRA1-60 (Millipore, MAB4360), 1:100; mouse anti-human NESTIN (Millipore, ABD69), goat anti-SOX2 (Santa Cruz sc-17320), 1:200; rabbit anti-βIII-tubulin/Tuj 1 (Covance PRB-435P), 1:200; mouse anti-MAP2AB (Sigma M1406), 1:200. Secondary antibodies were Alexa donkey anti-rabbit 488 (Jackson Immuno 711-545-152) and 568(Life Technologies A10042), Alexa donkey anti-mouse 488 (Jackson Immuno 715-545-151) and 568 anti-mouse (Life Technologies A10037), and Alexa donkey anti-goat 488 (Jackson Immuno 705-545-147) and 568 (Jackson Immuno 705-605-147); all were used at 1:300.

IMMUNOHISTOCHEMISTRY (IHC): anti-chicken GFP (Abcam, 1:5000; ab13970).

Antibodies used for immunoprecipitation were: mouse IgG (Santa Cruz cat. # sc-2025), α-SMARCA4 (BRG1 H-10, mouse monoclonal, Santa Cruz cat. # sc-374197), α-SS18L1 (CREST M-15, goat polyclonal, Santa Cruz cat. # sc-50912), α-SS18L1#2 (CREST D-7, mouse monoclonal, Santa Cruz cat. # sc-515827), α-SMARCC2 (BAF170 E-6, mouse monoclonal, Santa Cruz cat. # sc-17838), α-SMARCB1 (INI1/BAF47 A-5, mouse monocolonal, Santa Cruz cat. # sc-166165), α-SMARCD3 (BAF60C RN-18, mouse monoclonal, Santa Cruz cat. # sc-101163; this antibody did not appear to work for IP). For immunoblotting, additional antibodies were used: α-HA (6E2, mouse monoclonal, Cell Signaling cat. # 2367; this antibody was by far the most clean of several α-HA antibodies tested), α-SMARCA2/4 (BRG1/BRM J1 clone, rabbit polyclonal, made in-house), α-ACTL6B/BAF53B (rabbit polyclonal, made in-house), α-TOP2B (F-12, rabbit polyclonal, Santa Cruz cat. # sc-365916), α-ARID1B (mouse monoclonal, Novus Biologicals cat. # H00057492-M01), α-PBRM1 (BAF180 D3F7O, rabbit monoclonal, Cell Signaling cat. #91894), α-TBP (mouse monoclonal, Abcam cat. # ab818), α-B-ACTIN (AC-15, mouse monoclonal, Santa Cruz cat. #sc-69879). Goat or donkey, α-mouse or α-rabbit IRDye 800CW or 680LT (LI-COR) secondary antibodies were used for Western blot analysis

Validation

Antibodies used in this study (all of which have been commercially validated by the manufacturers) were validated in human/rodent cells/tissues via immunoblotting, IPs or ICC/IHC/IF prior to experimentation (analyzed for correct migration size, sub-cellular localization patterns, etc.).

Animals and other research organisms

Policy information about studies involving animals; ARRIVE guidelines recommended for reporting animal research, and Sex and Gender in Research

Laboratory animals

All mice used in DS related studies exist on a mixed genetic background, with comparative groups (euploid vs. Brwd1+/– vs. Ts65Dn vs. Ts65Dn;Brwd1+/–) maintained on the same mixed background (DBA/2J x B6EiC3Sn/J) for experimental testing. Briefly, trisomic Ts (176)65Dn (Ts65Dn) females (Jackson Labs Stock 001924) were crossed to B6EiC3Sn/J (Stock 001875) euploid males to generate euploid and Ts65Dn animal littermates for initial Brwd1 expression analyses. Brwd1repro5 mutant mice (Mouse Genome Informatics [MGI] ID 3512929)14 were kindly provided by Dr. John Schimenti (Cornell). Heterozygotic Brwd1repro5 mutant male mice, aka Brwd1 +/– (fully backcrossed to B6EiC3Sn/J to match the breeding scheme for euploid vs. Ts65Dn animals), were crossed to Ts65Dn females to generate euploid vs. Brwd1+/– vs. Ts65Dn vs. Ts65Dn;Brwd1+/– animals for genetic rescue experiments. Given that suitable antibodies for the detection of endogenous Brwd1 in rodent tissues are not commercially available, we generated Brwd1FLAG-HA mice for in vivo interrogations.

Mice were group housed (separated by sex) – with the exception of surgerized animals, which were singly housed post-surgery –

	under a 12-h-light/dark cycle at constant temperature (25°C) and humidity (40-60%) with ad libitum access to food and water. Animals arriving from external sources were allowed at least one week of habituation to housing conditions prior to experimentation. Both male and female mice were assessed in these studies E16 primary cultures/E17.5 (mixed male and female) and adult (6-week, males and females analyzed separately).
Wild animals	The study did not involve samples collected from wild animals.
Reporting on sex	Both male and female mice were assessed in these studies [E16 primary cultures/E17.5 forebrain tissue (mixed male and female) and adult (6-week, males and females analyzed separately).
Field-collected samples	The study did not involve samples collected from the field.
Ethics oversight	All procedures were performed in accordance with NIH guidelines and the Institutional Animal Use and Care Committees (IACUC) at the Icahn School of Medicine at Mount Sinai, Michigan State University and Cornell University.

Note that full information on the approval of the study protocol must also be provided in the manuscript.

ChIP-seq

Data deposition

- [x] Confirm that both raw and final processed data have been deposited in a public database such as GEO.
- [x] Confirm that you have deposited or provided access to graph files (e.g. BED files) for the called peaks.

Data access links
May remain private before publication.

Data from RNA-seq, ChIP-seq and ATAC-seq experiments have been deposited in the National Center for Biotechnology Information Gene Expression Omnibus (GEO) database under accession numbers GSE210117 and GSE151255.

GEO Reviewer token:
GSE151255 (token = khadkasstvcvlct)
GSE210117 (token = mzspgqmmjletdwh)

Files in database submission

Raw and processed files (including bigWig files) are included for each of the samples listed below:

E16_FB-derived_neuronal_culture_RNA-Seq_Ts65Dn; Brwd1+/– _1
E16_FB-derived_neuronal_culture_RNA-Seq_Ts65Dn; Brwd1+/– _2
E16_FB-derived_neuronal_culture_RNA-Seq_Ts65Dn; Brwd1+/– _3
E16_FB-derived_neuronal_culture_RNA-Seq_Euploid _1
E16_FB-derived_neuronal_culture_RNA-Seq_Euploid _2
E16_FB-derived_neuronal_culture_RNA-Seq_Euploid _3
E16_FB-derived_neuronal_culture_RNA-Seq_Ts65Dn_1
E16_FB-derived_neuronal_culture_RNA-Seq_Ts65Dn_2
E16_FB-derived_neuronal_culture_RNA-Seq_Ts65Dn_3

EU1_10813_4_S16_R1
EU1_10813_4_S16_R2
EU2_10813_5_S17_R1
EU2_10813_5_S17_R2
EU3_10813_6_S18_R1
EU3_10813_6_S18_R2
EU4_10813_8_S19_R1
EU4_10813_8_S19_R2
EU5_10795_1_S20_R1
EU5_10795_1_S20_R2
EU6_10795_2_S21_R1
EU6_10795_2_S21_R2
EU7_10795_4_S23_R1
EU7_10795_4_S23_R2
EU8_10795_7_S25_R1
EU8_10795_7_S25_R2
TS1_10813_1_S13_R1
TS1_10813_1_S13_R2
TS2_10813_2_S14_R1
TS2_10813_2_S14_R2
TS3_10813_3_S15_R1
TS3_10813_3_S15_R2
TS4_10795_3_S22_R1
TS4_10795_3_S22_R2
TS5_10795_6_S24_R1
TS5_10795_6_S24_R2

TS6_10795_10_S26_R1
TS6_10795_10_S26_R2
TS7_10795_11_S27_R1
TS7_10795_11_S27_R2

Galaxy146-[F_CR__F_CR_B113]
Galaxy146-[F_CR__F_CR_B133]
Galaxy146-[F_CR__F_CR_B136]
Galaxy146-[F_CR__F_CR_B166]
Galaxy146-[F_CR__F_CR_B167]
Galaxy146-[F_CR__F_CR_B175]
Galaxy146-[F_CR__F_CR_B176]
Galaxy146-[F_CR__F_CR_B181]
Galaxy150-[M_TS__M_TS_B145]
Galaxy150-[M_TS__M_TS_B25]
Galaxy150-[M_TS__M_TS_B40]
Galaxy150-[M_TS__M_TS_B45]
Galaxy150-[M_TS__M_TS_B52]
Galaxy150-[M_TS__M_TS_B74]
Galaxy150-[M_TS__M_TS_B88]
Galaxy170-[M_WT__M_WT_B100]
Galaxy170-[M_WT__M_WT_B110]
Galaxy170-[M_WT__M_WT_B122]
Galaxy170-[M_WT__M_WT_B139]
Galaxy170-[M_WT__M_WT_B79]
Galaxy170-[M_WT__M_WT_B80]
Galaxy170-[M_WT__M_WT_B84]
Galaxy170-[M_WT__M_WT_B95]
Galaxy179-[F_WT__F_WT_B12]
Galaxy179-[F_WT__F_WT_B125]
Galaxy179-[F_WT__F_WT_B17]
Galaxy179-[F_WT__F_WT_B173]
Galaxy179-[F_WT__F_WT_B184]
Galaxy179-[F_WT__F_WT_B21]
Galaxy179-[F_WT__F_WT_B85]
Galaxy179-[F_WT__F_WT_B65]
Galaxy204-[F_TS__F_TS_B103]
Galaxy204-[F_TS__F_TS_B134]
Galaxy204-[F_TS__F_TS_B135]
Galaxy204-[F_TS__F_TS_B53]
Galaxy204-[F_TS__F_TS_B56]
Galaxy204-[F_TS__F_TS_T139]
Galaxy204-[F_TS__F_TS_T141]
Galaxy204-[F_TS__F_TS_T142]
Galaxy376-[M_CR__M_CR_B137]
Galaxy376-[M_CR__M_CR_B138]
Galaxy376-[M_CR__M_CR_B24]
Galaxy376-[M_CR__M_CR_B26]
Galaxy376-[M_CR__M_CR_B51]
Galaxy376-[M_CR__M_CR_B69]
Galaxy376-[M_CR__M_CR_B94]
Galaxy376-[M_CR__M_CR_T35]

Adult_Hippocampus_Male_Smarca2/4 ChIP-Seq_Euploid_IP_1
Adult_Hippocampus_Male_Smarca2/4 ChIP-Seq_Euploid_IP_2
Adult_Hippocampus_Male_Smarca2/4 ChIP-Seq_Euploid_IP_3
Adult_Hippocampus_Male_Smarca2/4 ChIP-Seq_Ts65Dn_IP_1
Adult_Hippocampus_Male_Smarca2/4 ChIP-Seq_Ts65Dn_IP_2
Adult_Hippocampus_Male_Smarca2/4 ChIP-Seq_Ts65Dn_IP_3
Adult_Hippocampus_Male_Smarca2/4 ChIP-Seq_Ts65Dn_IP_4
Adult_Hippocampus_Male_Smarca2/4 ChIP-Seq_Ts65Dn; Brwd1+/–_IP_1
Adult_Hippocampus_Male_Smarca2/4 ChIP-Seq_Ts65Dn; Brwd1+/–_IP_2
Adult_Hippocampus_Male_Smarca2/4 ChIP-Seq_Ts65Dn; Brwd1+/–_IP_3
Adult_Hippocampus_Male_Smarca2/4 ChIP-Seq_Ts65Dn; Brwd1+/–_IP_4
Adult_Hippocampus_Male_Smarca2/4 ChIP-Seq_Euploid_IP_4
Adult_Hippocampus_Male_Smarca2/4 ChIP-Seq_Euploid_Input_1
Adult_Hippocampus_Male_Smarca2/4 ChIP-Seq_Euploid_Input_2

Adult_Hippocampus_Male_Smarca2/4 ChIP-Seq_Euploid_Input_3
Adult_Hippocampus_Male_Smarca2/4 ChIP-Seq_Ts65Dn_Input_1
Adult_Hippocampus_Male_Smarca2/4 ChIP-Seq_Ts65Dn_Input_2
Adult_Hippocampus_Male_Smarca2/4 ChIP-Seq_Ts65Dn_Input_3
Adult_Hippocampus_Male_Smarca2/4 ChIP-Seq_Ts65Dn_Input_4
Adult_Hippocampus_Male_Smarca2/4 ChIP-Seq_Ts65Dn; Brwd1+/–_Input_1
Adult_Hippocampus_Male_Smarca2/4 ChIP-Seq_Ts65Dn; Brwd1+/–_Input_2
Adult_Hippocampus_Male_Smarca2/4 ChIP-Seq_Ts65Dn; Brwd1+/–_Input_3
Adult_Hippocampus_Male_Smarca2/4 ChIP-Seq_Ts65Dn; Brwd1+/–_Input_4
Adult_Hippocampus_Male_Smarca2/4 ChIP-Seq_Euploid_Input_4
Adult_Hippocampus_Male_Neuronal ATAC-Seq_Ts65Dn; Brwd1+/– _1
Adult_Hippocampus_Male_Neuronal ATAC-Seq_Ts65Dn; Brwd1+/– _2
Adult_Hippocampus_Male_Neuronal ATAC-Seq_Ts65Dn; Brwd1+/– _3
Adult_Hippocampus_Male_Neuronal ATAC-Seq_Ts65Dn _1
Adult_Hippocampus_Male_Neuronal ATAC-Seq_Ts65Dn _2
Adult_Hippocampus_Male_Neuronal ATAC-Seq_Ts65Dn _3
Adult_Hippocampus_Male_Neuronal ATAC-Seq_Euploid _1
Adult_Hippocampus_Male_Neuronal ATAC-Seq_Euploid _2
Adult_Hippocampus_Male_Neuronal ATAC-Seq_Euploid _3

Genome browser session
(e.g. UCSC)

https://ramaka02.u.hpc.mssm.edu/Brg1_ChIPseq/
https://ramaka02.u.hpc.mssm.edu/Brg1_ATACseq/

Methodology

Replicates

For RNA-seq experiments in brain tissue, a n=7-8 biological replicates/group were used, with n=3/group used for E16 primary neuronal cultures. For all ChIP-seq and ATAC-seq experiments, a n=3-4 biological replicates were used in each group.

Sequencing depth

Average sequencing depth for RNA-seq experiments: 20M reads
Average sequencing depth for ChIP-seq experiments: 60M reads
Average sequencing depth for ATAC-seq experiments: 80M reads

Antibodies

Samples were then incubated with a custom anti-SMARCA2/4 antibody provided by the Crabtree lab (Reference #39).

Peak calling parameters

Peak-calling was performed using MACSv2.1.124 with default settings.

Data quality

Peaks were filtered for FDR < 0.05 and fold change > 1.2. All processed peak data, along with respective fold changes and FDR values, are provided in the Extended Data Tables as part of this initial submission.

Software

HISAT2
MACSv2.1.124
diffReps

ATAC-seq differential lists were compared to ChIP-seq differential lists based on gene names using geneOverlap (GeneOverlap: Test and visualize gene overlaps. R package version 1.23.0, http://shenlab-sinai.github.io/shenlab-sinai/), and heat maps were drawn using the deepTools package
